# Interventions to reduce pedestrian road traffic injuries: A systematic review of randomized controlled trials, cluster randomized controlled trials, interrupted time-series, and controlled before-after studies

**DOI:** 10.1371/journal.pone.0262681

**Published:** 2022-01-24

**Authors:** Stellah Namatovu, Bonny Enock Balugaba, Kennedy Muni, Albert Ningwa, Linda Nsabagwa, Fredrick Oporia, Arthur Kiconco, Patrick Kyamanywa, Milton Mutto, Jimmy Osuret, Eva A. Rehfuess, Jacob Burns, Olive Kobusingye

**Affiliations:** 1 School of Public Health, Makerere University Kampala Uganda, Kampala, Uganda; 2 Department of Epidemiology, University of Washington, Seattle, WA, United States of America; 3 School of Health Sciences, Kampala International University, Bushenyi, Uganda; 4 Institute for Medical Information Processing, Biometry and Epidemiology, Ludwig-Maximillians Universitaet (LMU Munich), Munich, Germany; Tsinghua University, CHINA

## Abstract

**Background:**

Road traffic injuries are among the top ten causes of death globally, with the highest burden in low and middle-income countries, where over a third of deaths occur among pedestrians and cyclists. Several interventions to mitigate the burden among pedestrians have been widely implemented, however, the effectiveness has not been systematically examined.

**Objectives:**

To assess the effectiveness of interventions to reduce road traffic crashes, injuries, hospitalizations and deaths among pedestrians.

**Methods:**

We considered studies that evaluated interventions to reduce road traffic crashes, injuries, hospitalizations and/or deaths among pedestrians. We considered randomized controlled trials, interrupted time-series studies, and controlled before-after studies. We searched MEDLINE, EMBASE, Web of Science, WHO Global Health Index, Health Evidence, Transport Research International Documentation and ClinicalTrials.gov through 31 August 2020, and the reference lists of all included studies. Two reviewers independently screened titles and abstracts and full texts, extracted data and assessed the risk of bias. We summarized findings narratively with text and tables.

**Results:**

A total of 69123 unique records were identified through the searches, with 26 of these meeting our eligibility criteria. All except two of these were conducted in high-income countries and most were from urban settings. The majority of studies observed either a clear effect favoring the intervention or an unclear effect potentially favoring the intervention and these included: changes to the road environment (19/27); changes to legislation and enforcement (12/12); and road user behavior/education combined with either changes to the road environment (3/3) or with legislation and enforcement (1/1). A small number of studies observed either a null effect or an effect favoring the control.

**Conclusions:**

Although the highest burden of road traffic injuries exists in LMICs, very few studies have examined the effectiveness of available interventions in these settings. Studies indicate that road environment, legislation and enforcement interventions alone produce positive effects on pedestrian safety. In combination with or with road user behavior/education interventions they are particularly effective in improving pedestrian safety.

## Introduction

Road traffic injuries (RTIs) are among the top ten causes of death for people of all ages across all regions [1]. The impact of RTIs is greater among people aged 5–29 years where it is the leading cause of death [2]. Between 2013 and 2016, reductions in the number of road traffic deaths were recorded among 48 middle- and high-income countries [1]. However, the road traffic burden remains predominant among pedestrians in low- and middle-income countries (LMICs), especially in the African, Eastern Mediterranean, and Western Pacific regions. Pedestrians in the African region account for 38% of the road traffic deaths, which is higher than their global contribution of 22%. Only examining deaths is likely to an underestimate of the true pedestrian burden, because minor and moderate pedestrian injuries are often unreported [3].

There are several interventions that have been widely implemented to mitigate the burden of pedestrian RTIs [4]. The implementation of these interventions and the capacity for their implementation vary across regions and countries. Following the Haddon Matrix Framework (Table 1), these interventions fall in the broad categories of host (behavior change), environment (road infrastructure), and agent (vehicles). The Haddon Matrix is relevant for understanding how interventions work and it also aids in the identification of interventions at the pre-crash, crash and post-crash phases [5].

**Table 1:** Haddon Matrix applied to pedestrian-impacting interventions.

	Pre-event (pre-crash)	Event (crash)	Post-event (post-crash)
**Aim**	Crash prevention	Injury prevention during crash	Mitigating effects of the crash
**Host (behavior)**	• Law and enforcement • Education and training • Driver training and licensing • Fitness to drive assessment • Awareness raising and campaigns Pedestrian regulation	First Aid	• Seeking care • Emergency medical services (pre-hospital care) • Good Samaritan laws • First responders/lay responders
**Agent (vehicle)**	• Vehicle inspection and roadworthiness • Vehicle maintenance (e.g., braking) • Automatic emergency braking	• Active Technology for Pedestrian Protection • Vehicle Shape for Pedestrian Protection	Ambulances Extraction from vehicle
**Physical & social environment (Infrastructure)**	• Speed management and enforcement • Visibility/lighting treatments • Road markings • Sidewalk treatments • Driver information alert • Traffic signs treatments	• Forgiving road environments	• Rehabilitation infrastructure and infrastructure for integration in society/work after crash • Triage and allocation to trauma facilities

There is increasing pressure for LMICs to invest in interventions to reduce pedestrian injuries. Road safety was included under the Sustainable Development Goals (Goal 3.6 & 11.2) to reduce global road deaths by at least 50% through the provisioning of safe transport systems [6]. Most high-income countries (HICs) have reduced road traffic deaths over the last few decades, but their road environments, population structures, and transport systems are markedly different from those of low-income countries (LICs) where the burden is currently the highest. There is a paucity of systematically pooled evidence on effective interventions that could potentially reduce pedestrian injuries, especially in the countries with the highest pedestrian injury rates. The aim of this systematic review was to assess the effectiveness of interventions to reduce road traffic crashes, injuries, hospitalizations and deaths among pedestrians.

## Methods

### Protocol and registration

This systematic review was conducted according to the Cochrane guidelines and reported according to the PRISMA statement [7] see S1 Checklist. We registered the protocol (in the International Prospective Register of Systematic Reviews (PROSPERO) database under the number CRD42018104287 (see S1 Text).

Ethical approval: The Higher Degrees, Research and Ethics Committee of Makerere University School of Public Health and the Uganda National Council for Science and Technology approved this systematic review which is embedded in a bigger project titled, “Finding the evidence for improved implementation of road safety interventions to reduce pedestrian injuries and deaths in Uganda”. For the systematic review, as only existing publications were examined, we did not require obtaining written or verbal consent.

### Eligibility criteria

We included pedestrians of all ages and excluded cyclists, skaters, scooter drivers, bicyclists, those taking part in motorized or vehicle traffic (automobile drivers and passengers, public transport riders, motorcyclists). We also excluded all studies that did not disaggregate results by road user type i.e., where data on pedestrians and drivers or other road users were combined as shown in S1 Table. We assessed studies that involved any of the four following broad categories of road safety interventions: road environment, road user behavior, legislation and enforcement and vehicle design and road worthiness status. Regarding the comparator, we considered studies comparing the intervention to no intervention, as well as to other road traffic interventions. We focused on studies that assessed the following four outcomes: pedestrian crashes, pedestrian injuries, pedestrian hospitalizations and/or pedestrian deaths. We included randomized controlled trials (RCTs), cluster randomized controlled trials (c-RCTs), controlled before-after (CBA) and both uncontrolled and controlled interrupted time-series (uITS and cITS respectively).

### Search strategy

Our search strategy was based on “population”, “intervention”, “outcome” and “study design” search blocks. The search strategy was developed iteratively by the review team, with each version being tested against a set of test studies eligible for inclusion and an initial screening of 100 titles and abstracts. The Ovid strategy was applied for MEDLINE and EMBASE and adapted to all other databases, provided in S1 Table.

### Data sources

We searched the following electronic databases MEDLINE, EMBASE, Web of Science, WHO Global Health Index, Health Evidence, Transport Research International Documentation (TRID) and ClinicalTrials.gov up to 31 August 2020.We considered all original research studies that met the criteria, published in English, German and Spanish regardless of their publication status and with no restriction on the year of publication. One review team member who speaks German and Spanish reviewed the studies in those two languages. We further screened the reference lists of the included studies for more studies to include.

### Study selection

Each unique record was reviewed at the title/abstract stage by two independent reviewers to determine eligibility. Any disagreements between the two reviewers were discussed and resolved by consensus and where necessary, a third reviewer was consulted. For all studies deemed potentially eligible or unclear at the title and abstract screening stage, we retrieved the full texts, and these were assessed for eligibility, analogous to the process described above. We used EndNote software to manage retrieved studies and remove duplicate reports, and Rayyan, a web-based application, to manage the title and abstract screening process.

### Data extraction

For all studies meeting the inclusion criteria, two reviewers used the data collection form to extract data on study designs, study population, characteristics of the intervention and comparison groups, statistical analysis, outcomes and effects. Where important data were not available to extract (e.g., related to outcomes and/or effects) we contacted authors at least twice requesting the relevant data.

### Risk of bias assessment

Given the diversity in the designs of the studies included, we used the Cochrane ‘Risk of bias’ tool [7] as modified by Cochrane EPOC to assess risk of bias [8]. Separate criteria were applied to ITS studies and non-ITS studies. For non-ITS studies, assessed domains included i) baseline outcome measurements ii) other baseline characteristics, iii) incomplete outcome data, iv) blinding, v) study contamination, vi) selective outcome reporting and vii) other possible risks of bias. Domains i, ii and v were not applied to uITS studies, but other assessed domains included viii) independence of the intervention from other changes, ix) pre-specification of the intervention effect and x) intervention effects on data collection. For cITS studies all domains were assessed. The risk of bias for each study was assessed by 2 independent reviewers and then consensus reached after discussion. For each domain, we assigned low, unclear, or high risk of bias as summarized in S2 Table.

### Data synthesis

Based on the methodological heterogeneity of the included studies in terms of the study designs, analytical methods, outcomes, and the reporting of the results, we deemed meta-analysis to be inappropriate. Instead, we conducted a synthesis without meta-analysis (SWiM), which comprised a narrative synthesis as well as a graphical summary, based on the method of vote counting. In doing so, we adhered to recent SWiM guidance and the Cochrane Handbook [9,10].

The key to this method involved defining the effect direction for the main effect(s) reported in each included study. Using the guidance cited above, as well as recently published Cochrane systematic reviews [11–13], we classified the effect(s) for each intervention category-outcome pair from each included study as one of the following effect directions:

- *Clear effect favoring the intervention*: effect in the direction of the intervention, with clear indication that the 95% confidence interval does not contain the null, or p-value < 0.05.
- *Unclear effect potentially favoring the intervention*: effect in the direction of the intervention, however either the 95% confidence interval contains the null effect, p-value > 0.05, or no direct comparison was provided (e.g., separate analyses assessing changes at intervention and control sites, yet the two are not formally compared with one another).
- *Null effect*: no difference seen between the intervention and control. Studies only narratively reporting that ‘no significant effect was observed’ between intervention and control were classified here, however studies reporting an actual effect estimate were only classified here if a true null effect was reported.
- *Unclear effect potentially favoring the control*: effect in the direction of the control, however either the 95% confidence interval contains the null effect, p-value > 0.05, or no direct comparison was provided.
- *Clear effect favoring the control*: effect in the direction of the control, with clear indication that the 95% confidence interval does not contain the null, or p-value < 0.05.

This facilitates the synthesis of heterogeneous bodies of evidence such as this one, where it is deemed either impossible or inappropriate to pool effect estimates from individual studies. As there is no widely accepted minimally important difference for road traffic interventions to reduce pedestrian injuries, we consider any difference from the null in classifying effect direction.

In narratively and graphically summarizing the effect directions, we stratified the results based on the priori defined intervention and outcome categories.

### Assessment of certainty of evidence

We used the Grades of Recommendation, Assessment, Development and Evaluation (GRADE) system to assess the certainty of evidence [14]. As per GRADE guidance, bodies of evidence comprising randomized studies started the assessment at high certainty, those comprising non-randomized studies started the assessment at low certainty. For each identified body of evidence (i.e., intervention category-outcome pair) we then assessed study limitations, inconsistency, imprecision, indirectness and publication bias to downgrade the body of evidence, where applicable, and considered three criteria for upgrading (i.e., large magnitude of effect, dose-response gradient, plausible confounding leading to underestimate of effect). Based on these aspects, each body of evidence was ultimately rated as very low, low, moderate or high.

## Results

### Study selection and description of included studies

**Fig 1:**
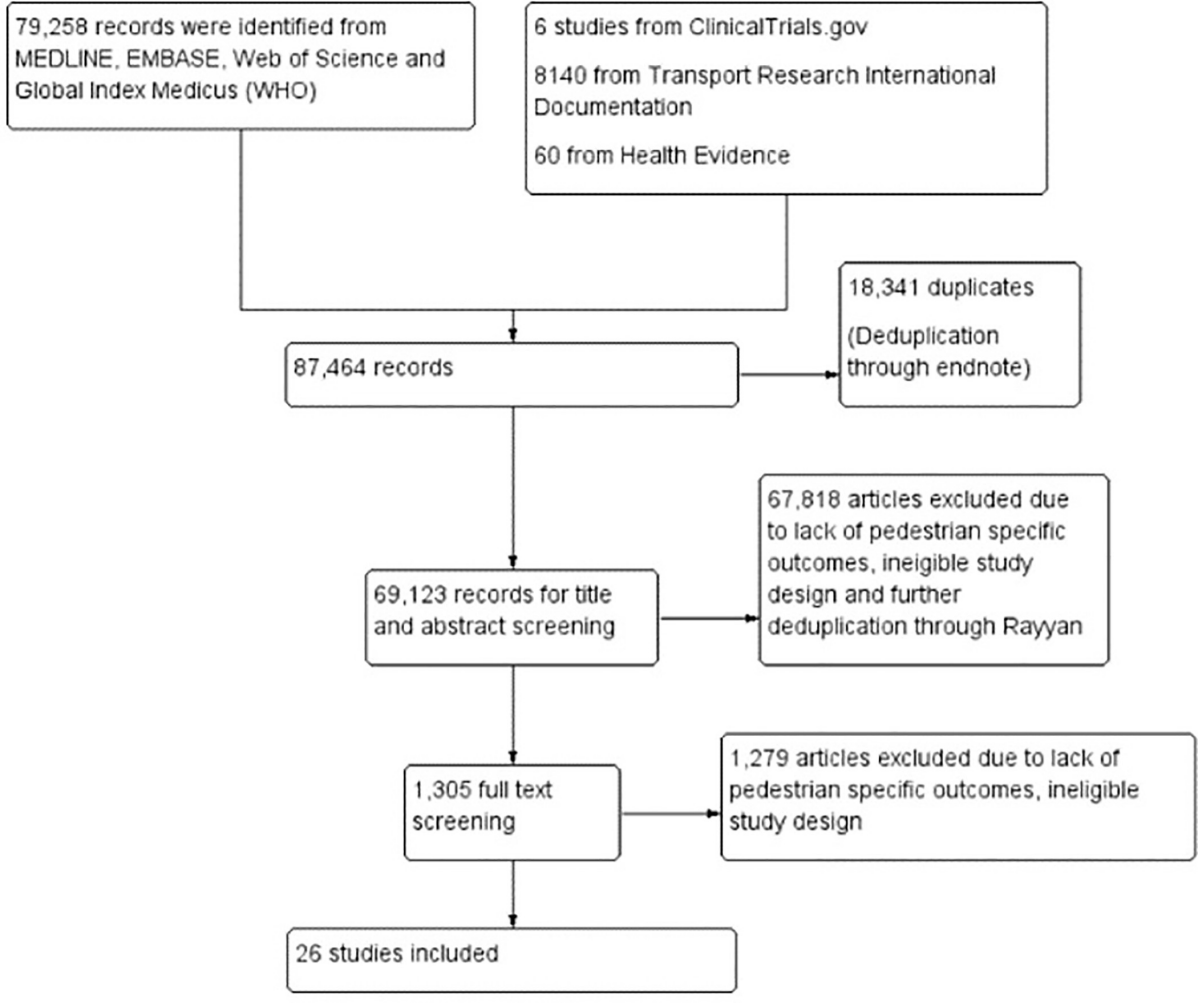
Flowchart summarizing the search and study selection process.

After de-duplication, our search strategy returned a total of 69123 unique records. Through title and abstract screening, we deemed 1350 of these potentially relevant; after full text screening, 26 studies met our eligibility criteria. This process is illustrated in Fig 1.

The 26 included studies are described in Table 2, stratified by intervention category. All but two of the included studies were conducted in HICs and most of the interventions were carried out in urban settings. Regarding the population of interest, most studies (22) drew from routine data sources, and thus investigated pedestrian safety across the general population; two studies assessed data collected from households, while for two studies the nature of the data source was unclear.

**Table 2:** Summary of characteristics of included studies.

Study ID	Country	Study design/duration (years)	Intervention description	Outcome measure
**Road environment changes**				
Chen 2013 [15]	USA	CBA (18)	Installed pedestrian phase and increased pedestrian crossing time	Pedestrian crashes^1^
Chen 2014 [16]	USA	CBA (7)	Increasing cycle length, Barnes dance, split-phase timing, signal installation	Pedestrian crashes^1^
Choi 2018 [17]	South Korea	CBA (8)	Silver zone in lowering the number of elderly pedestrian-vehicular collisions	Pedestrian crashes^1^
Dimaggio 2013 [18]	USA	CBA (10)	Implementation of a Safe Routes to School program	Pedestrian injuries^1^
Ewing 2013 [19]	USA	CBA (10)	Construction of speed tables	Pedestrian crashes^3^
Feldman 2010[20]	USA	CBA (12)	Converted the markings on all standard, yellow school crosswalks at 900 locations to high-visibility, yellow, continental-style striping and replaced yellow school-warning signs with fluorescent yellow-green warning signs throughout the city.	Pedestrian crashes^1^
Naznin 2016 [21]	Australia	CBA (not reported)	Platform tram stops constructed progressively to replace older design stops.	Pedestrian crashes ^1^
Noland 2007[22]	Britain	uITS (13)	Area-based congestion charging scheme was implemented in the central area of London.	Pedestrian injuries and deaths^1^
Olsen 2016 [23]	United Kingdom	CBA (17)	Construction of new motor way	Pedestrian injuries^1^
Persaud 1997 [24]	USA	CBA (14)	Converting one-way street intersections from signal to multiway stop sign control. (Signal Removal)	Pedestrian crashes and injuries^3^
Polus1978 [25]	Israel	CBA (5)	Illuminated cross walk to warn drivers of a pedestrian.	Pedestrian crashes^1^
Rothman 2017 [26]	Canada	CBA (3)	Installation of pedestrian countdown signals (PCS) at intersections.	Pedestrian crashes^1^
Song2019 [27]	USA	c-ITS (15)	Improved transit operational performance through adjusting signal timing and providing approaching transit vehicles longer or earlier green lights.	Pedestrian crashes^1^
Steinbach 2010 [28]	United Kingdom	CBA (20)	20 mph zones with traffic calming measures such as speed humps and chicanes.	Pedestrian injuries and deaths^1^
Yamanaka 1998 [29]	Japan	CBA (16)	Traffic calming measures including intersection humps, entry narrowing and raised junction.	Pedestrian crashes^1^
Yannis 2014 [30]	Greece	CBA (14)	Traffic calming measures implemented including: speed humps, raised intersections, curb extensions, road closures and woonerfs.	Pedestrian crashes^1^
**Legislation and enforcement**				
Green2014 [31]	United Kingdom	CBA (8)	Free bus travel scheme for young people with unlimited free bus travel.	Pedestrian injuries^1^
Kloeden 2006 [32]	Australia	CBA (11)	Legislation to decrease the speed limit to 50 km/h	Pedestrian crashes^1^
Kweon 2009 [33]	USA	u-ITS (25)	Motorists stop, rather than yield, for pedestrians at cross walks	Pedestrian crashes^1^
Novoa 2011 [34]	Spain	u-ITS (9)	Criminalizing traffic offences. Penal code modified to criminalize several traffic offenses.	Pedestrian injuries^1^
Porter 2018 [35]	USA	c-ITS (38)	Legislating road design to provide for pedestrians and cyclists	Pedestrian crashes^1^
Preusser 1982 [36]	USA	u-ITS (not indicated)	Permissive Right Turn on Red	Pedestrian crashes^1^
**Road user behavior/education interventions**				
Zimmerman 2015 [37]	Tanzania	CBA (0.75)	Implemented a road safety intervention program. Training, reflector vests, and education for school children and calendars displaying road safety messages to community members	Pedestrian injuries^2^
**Combined interventions**				
Agent 1996 [38]*	USA	CBA (6)	Implementation of a regional traffic safety improvement program to increase safety belt usage, and to increase public awareness of the effects of alcohol-related and high-speed driving in rural areas."	Pedestrian crashes, injuries and deaths^1^
Durkin 1999 [39] **	USA	u-ITS (7)	Implementation of a comprehensive, hospital-initiated injury prevention program. safety city traffic education program for elementary school children and building of play grounds and initiation of adult mentoring	Pedestrian injuries and deaths^1^
Poswayo 2018 [40] **	Tanzania	CBA (1.3)	Infrastructure enhancements designed to lower vehicle speeds and separate pedestrians from traffic such as speed bumps, rumble strips, bollards, moving school gates, zebra crossings, sidewalks and accompanying signage as well as a road safety education programme tailored to the school and advocacy to local and national level government.	Pedestrian Injuries ^2^

* Road user behavior/education and Legislation and Enforcement.

** Road user behavior/education and road environment.

^1^Routinely collected data.

^2^Household data.

^3^Unclear source of data.

Abbreviations: CBA: Controlled Before-After study, uITS: uncontrolled Interrupted time-series study, cITS: controlled interrupted time-series study, RCT: Randomized controlled trial.

Sixteen studies investigated measures regarding the road environment, six investigated the legislation and enforcement, and one investigated road user behavior/education interventions while three other studies assessed interventions belonging to multiple categories, with two assessing both road user behavior/ education and road environment interventions, and one assessing road user behavior/education and legislation and enforcement interventions. Within these broader categories, however, a range of specific interventions were assessed. Changes to the road environment ranged for example, from the introduction of speed tables [15] and of countdown signals at intersections [16,26] to the construction of a new motorway [23]. Legislation and enforcement interventions included, among others, free bus travel for youth [27], reduction of speed limits [32] and stricter criminalization of traffic offenses [34]. The included road user behavior/education intervention [37] and the combined interventions [37–40] each comprised larger package interventions comprising multiple components.

Regarding the outcomes assessed in included studies, most studies assessed data on crashes, while a much smaller number of studies looked further at injuries and/or deaths. With regard to the study design, we identified nineteen CBA studies, four uITS studies and three cITS studies.

### Risk of bias of studies

**Fig 2:**
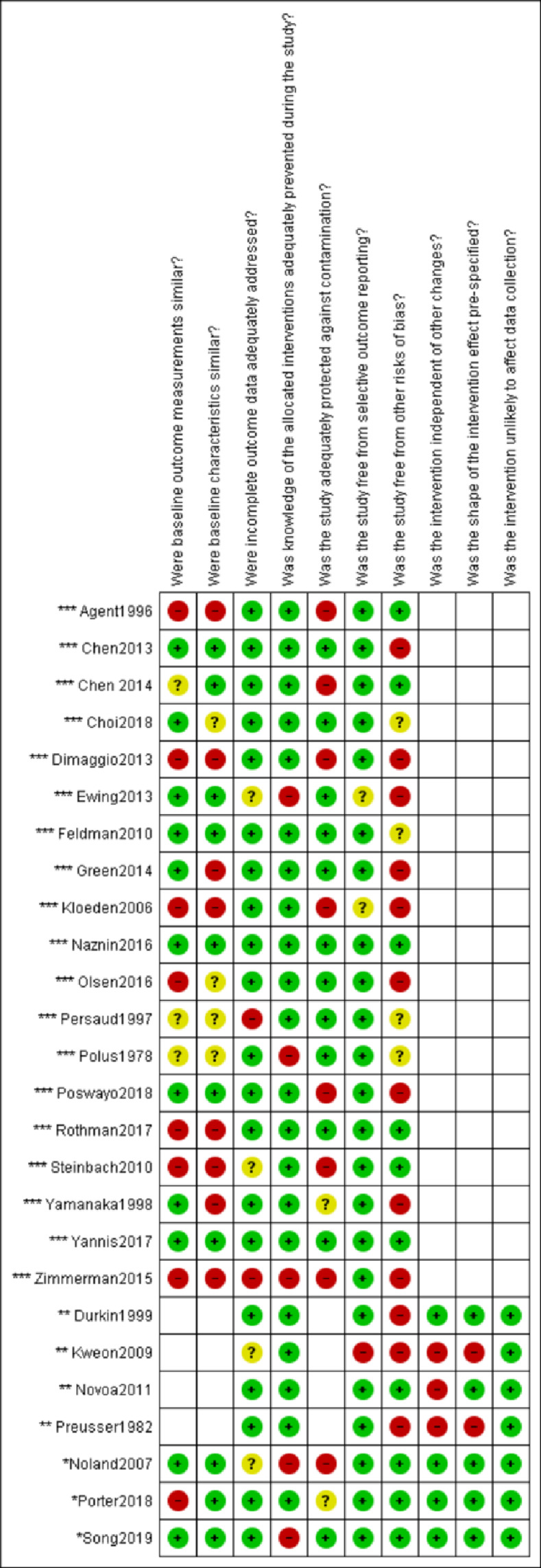
Summary of risk of bias of included studies. Different domains were applied to different study designs. * cITS study; ** uITS study, ***CBA study.

For CBA studies, common risks of bias were unbalanced groups with regards to baseline outcome measurements and other baseline characteristics, and inadequate protection against contamination. For several uITS studies, it was unclear whether the intervention was independent of other changes occurring. Overall, cITS studies had fewer concerns related to knowledge of the allocated prevented, baseline outcomes measurements and study not adequately protected against contamination. For most of the studies we identified other risks of bias, including, for example, interventions not being independent of other changes in the environment that could have affected the outcome [28,31,41] (Fig 2).

### Effectiveness of interventions

Effects from included studies for each intervention category-outcome pair, in the form of effect direction, are summarized in Table 3; the underlying effect estimates informing these effect directions are given in Table 4. Examining the evidence base holistically, a few aspects can be observed.

**Table 3:** Summary of effect directions, stratified by intervention category and outcome.

Outcomes	Clear effect favoring the intervention	Unclear effect potentially favoring the intervention	Null effect	Unclear effect potentially favoring the control	Clear effect favoring the control	Certainty of evidence*
**Changes to the road environment**						
Pedestrian crashes	↑ ^10^ ↑ ^10^ ↑ ^10^ ↑ ^15^ ↑ ^16^ ↑ ^16^	↗ ^10^ ↗ ^10^ ↗ ^10^ ↗ ^14^ ↗ ^19^ ↗ ^20^ ↗ ^22^ ↗ ^24^ ↗ ^25^	-	↘ ^10^ ↘ ^10^ ↘ ^10^ ↘ ^12^ ↘ ^21^ ↘ ^22^	-	Very low^a^
Pedestrian injuries	-	↗ ^13^ ↗ ^19^ ↗ ^19^		-	↓ ^18^	Very low^a^^,^ ^b^
Pedestrian deaths	-	-	-	-	-	-
Pedestrian injuries and deaths	-	↗ ^23^	◼^17^	-	-	Very low^b^
**Changes to legislation and enforcement**						
Pedestrian crashes	↑ ^31^ ↑ ^31^ ↑ ^31^ ↑ ^31^	↗ ^27^ ↗ ^28^ ↗ ^28^ ↗ ^28^	-	-	-	Very low^b^
Pedestrian injuries		↗ ^26^	-	-	-	Very low^c^
Pedestrian deaths	↑ ^30^	-	-	-	-	Low
Pedestrian injuries and deaths	↑ ^29^	↑ ^29^	-	-	-	Low
**Road user behavior/education interventions**						
Pedestrian crashes	-	-	-	-	-	-
Pedestrian injuries	-	-	-	↘ ^32^	-	Very low^c^
Pedestrian deaths	-	-	-	-	-	-
Pedestrian injuries and deaths	-	-	-	-	-	-
**Combined: Road user behavior/education interventions and changes to road environment**						
Pedestrian crashes	-	↗ ^35^	-	-	-	Very low^c^
Pedestrian injuries	↑ ^34^					Low
Pedestrian deaths	-	↗ ^34^	-	-	-	Low
Pedestrian injuries and deaths	-	-	-	-	-	-
**Combined: Road user behavior/education interventions and changes to legislation and enforcement**						
Pedestrian crashes	-	↗ ^33^	-	-	-	Very low^c^
Pedestrian injuries	-	-	-	-	-	-
Pedestrian deaths	-	-	-	-	-	-
Pedestrian injuries and deaths	-	-	-	-	-	-

Symbols represent the effect direction: (↑): clear effect favoring the intervention; (↗): unclear effect potentially favoring the intervention; (◼): null effect; (↘): unclear effect potentially favoring the control; (↓): clear effect favoring the control.

*As assessed by GRADE. Footnotes below:

^a^Downgraded -1 due to inconsistency, as studies observed effects favoring both the intervention and control.

^b^Downgraded -1 due to study limitations, as multiple studies are at high risk of bias across multiple domains.

^c^Downgraded -1 due to study limitations, as the only study contributing data is at high risk of bias across multiple domains.

**Table 4:** Data from included studies underlying the summary of effect direction.

Outcome	Study	Study design	Intervention	Effect summary	Effect direction
**Road environment changes**					
Pedestrian crashes	Chen 2013 [15]	CBA	1. Pedestrian phase 2. Split phase timing 3. Increasing pedestrian cross time 4. Pedestrian fencing 5. Signal installation 6. High visibility crosswalk 7. Speed limit reduction 8. Speed hump 9. Road diet	% change at intervention sites, relative to control sites 1: 35%; p < 0.05 2: -25%; p > 0.05 3: -51%; p < 0.05 4: 18%; p > 0.05 5: 1%; p > 0.05 6: -48%; p < 0.05 7: -35%; p > 0.05 8: 8%; p > 0.05 9: 41%; p > 0.05	1. ↑ 2. ↗ 3. ↑ 4. ↘ 5. ↘ 6. ↑ 7. ↗ 8. ↘ 9. ↘
	Chen 2014 [13]	CBA	1.Increasing cycle length 2. Barnes dance 3. Split-phase timing 4. signal installation	Study presents Average pedestrian crashes (per intersection per year) for each intervention type: 1. 0.23 2. 0.32 3. 0.75 4. 0.07	1. ↑ 2. ↑ 3. ↑ 4. ↗
	Rothman 2017 [26]	CBA	Installation of pedestrian countdown signals (PCS) at intersections	% change pre-intervention to post-intervention: Int: -1% Con: -22%	↘
	Feldman 2010 [20]	CBA	Converted the markings on all standard, yellow school crosswalks at 900 locations to high-visibility, yellow, continental-style striping and replaced yellow school-warning signs with fluorescent yellow-green warning signs throughout the city.	% change pre-intervention to post-intervention at intervention sites, relative to control sites: -37% (95% CI: 13%; 60%)	↑
	Polus 1978 [25]	CBA	Illuminated cross walk to warn drivers of a pedestrian	% change pre-intervention to post-intervention: Night-time (effect expected): -57% Day-time (no effect expected): -21% Control: 60%	↗
	Yamanaka 1998 [25]	CBA	Traffic calming measures including intersection humps, entry narrowing and raised junction.	Adj % change pre-intervention to post-intervention: Int: -18.9% Con: -2.1%	↗
	Song 2019 [25]	cITS	Improved transit operational performance through adjusting signal timing and providing approaching transit vehicles longer or earlier green lights.	Change in crashes per 100 miles travelled pre-intervention to post-intervention at intervention sites, relative to control sites: 1. Immediate change: -1.46 (95% CI: -4.93; 2.01) 2. Slope change: 0.06 (95% CI: 0.00; 0.12)	1. ↗ 2. ↘
	Choi 2018 [17]	CBA	Introduction of silver zones, which involve various structural changes near facilities for the elderly	Poisson DiD estimator expressing change pre-intervention to post-intervention at intervention sites, relative to control sites: 0.032; p > 0.05	↘
	Ewing 2013 [19]	CBA	Construction of speed tables	Adj change pre-intervention to post-intervention: -0.017; p = 0.51	↗
	Yannis 2014 [30]	CBA	Traffic calming measures implemented including: speed humps, raised intersections, curb extensions, road closures and woonerfs.	Change pre-intervention to post intervention Int: 9 crashes to 7 crashes Con: 26 crashes to 26 crashes; p > 0.05	↗
	Nanzin 2016 [21]	CBA	Platform tram stops constructed progressively to replace older design stops	% change pre-intervention to post-intervention at intervention sites, relative to control sites: 1. All crashes: -81%; p < 0.05 2. Serious and fatal crashes: -86%; p < 0.05	1. ↑ 2. ↑
	Persuad 1997 [24]	CBA	Converting one-way street intersections from signal to multiway stop sign control. (Signal Removal)	% change pre-intervention to post-intervention at intervention sites, relative to control sites: -18%	↗
Pedestrian injuries	DiMaggio 2013 [18]	CBA	Implementation of a Safe Routes to School program	% change pre-intervention to post-intervention: Int: -44% (95% CI: -65%; -17%); Con: 0% (-8%; 8%)	↗
	Olsen 2016 [23]	CBA	Construction of new motorway, which defers traffic away from residential area	% change pre-intervention to post-intervention: Int: -52% Con: -69% to -65% p < 0.05	↓
	Persuad 1997 [24]	CBA	Converting one-way street intersections from signal to multiway stop sign control. (Signal Removal)	% change pre-intervention to post-intervention at intervention sites, relative to control sites: 1. Minor injuries: -24.1% 2. Severe injuries: -54.5%	1. ↗ 2. ↗
Pedestrian injuries and deaths*	Noland 2007 [22]	uITS	Area-based congestion charging scheme was implemented in the central area of London.	Change pre-intervention to post intervention Specific data not reported for pedestrian outcomes: “note that pedestrian models are omitted as we found no significant effect on pedestrian casualties from the intervention” p > 0.05	◼
	Steinbach 2010 [28]	cITS	20 mph zones with traffic calming measures such as speed humps and chicanes.	% change pre-intervention to post intervention: Int (deprivation quintile 1): -35.1% Int (deprivation quintile 5): -30.9% Con (deprivation quintile 1): -24.1% Con (deprivation quintile 5): -4.4%	↗
**Legislation and enforcement changes**					
Pedestrian crashes	Kloeden 2006 [32]	CBA	Legislation to decrease the speed limit to 50 km/h	% change pre-intervention to post intervention Int: -20.6% Con: -17.8%	↗
	Kweon 2009 [33]	uITS	Motorists stop, rather than yield, for pedestrians at cross walks	% change pre-intervention to post intervention Int (Georgia): -0.9% Int (Minnesota): -0.9% Int (Washington): 2.0%	1. ↗ 2. ↗ 3. ↗
	Preusser 1982 [36]	uITS	Permissive Right Turn on Red	% change pre-intervention to post intervention Int (New York): 43%; p < 0.05 Int (Wisconson): 107%; p < 0.05 Int (Ohio): 57%; p < 0.05 Int (New Orleans): 82%; p < 0.05	1. ↑ 2. ↑ 3. ↑ 4. ↑
Pedestrian injuries	Green 2014 [31]	CBA	Free bus travel scheme for young people with unlimited free bus travel	% change pre-intervention to post-intervention: Int (age group 12–17 years): -29% Con (age group 60 years and older): -28%	↗
Pedestrian deaths	Porter 2018 [35]	cITS	Legislating road design to provide for pedestrians and cyclists	% change pre-intervention to post-intervention at intervention sites, relative to control sites: -0.5%; (95% CI: -0.954; -0.043)	↑
Pedestrian injuries and deaths*	Novoa 2011 [34]	uITS	Criminalizing traffic offences. Penal code modified to criminalize several traffic offenses	% change pre-intervention to post-intervention: Int (women): 10.7%; p < 0.05 Int (men): 8.3%; p > 0.05	1. ↑ 2. ↗
**Road user behavior/education interventions**					
Pedestrian injuries	Zimmerman 2015 [37]	CBA	Implemented a road safety intervention program. Training, reflector vests, and education for school children and calendars displaying road safety messages to community members	% change pre-intervention to post-intervention: Int: -28% Con: -58.7%	↘
**Combined: Road user behavior/education and road environment changes**					
Pedestrian crashes	Poswayo 2019 [35]	CBA	Infrastructure enhancements designed to lower vehicle speeds and separate pedestrians from traffic such as speed bumps, rumble strips, bollards, moving school gates, zebra crossings, sidewalks and accompanying signage as well as a road safety education programme tailored to the school and advocacy to local and national level government.	Change pre-intervention to post-intervention Int: -0.36 injuries per 100 person-years Con: 0.38 injuries per 100 person-years	↗
Pedestrian injuries	Durkin 1999 [34]	uITS	Implementation of a comprehensive, hospital-initiated injury prevention program. safety city traffic education program for elementary school children and building of play grounds and initiation of adult mentoring.	% change pre-intervention to post intervention: Int: -45%; (95% CI: -62%; -21%)	↑
Pedestrian deaths	Durkin 1999 [34]	CBA	Implementation of a comprehensive, hospital-initiated injury prevention program. safety city traffic education program for elementary school children and building of play grounds and initiation of adult mentoring.	Change pre-intervention to post intervention: Int (age group 5–16 years): -1.7 deaths/100000/year Con (age group <5 years): 0.7 deaths/100000/year	↗
**Combined: Road user behavior/education and legislation and enforcement**					
Pedestrian crashes	Agent 1996 [33]	CBA	Implementation of a regional traffic safety improvement program to increase safety belt usage, and to increase public awareness of the effects of alcohol-related and high-speed driving in rural areas.	% change pre-intervention to post intervention: Int: -51.3% Con: -19.3%	↗

_Symbols represent the effect direction: (↑): clear effect favoring the intervention; (↗): unclear effect potentially favoring the intervention; (◼): null effect; (↘): unclear effect potentially favoring the control; (↓): clear effect favoring the control. *Aggregate count of injuries and deaths._

Firstly, while a range of effect directions were observed across studies, a large majority of observed effects were classified as either a clear effect favoring the intervention (↑) or an unclear effect potentially favoring the intervention (↗). Specifically, the proportions of effects in one of these categories were 19/27 for changes to the road environment, 12/12 for changes to legislation and enforcement, 0/1 for road user behavior/education interventions, and 3/3 and 1/1 for road user behavior/education interventions combined with changes to the road environment and combined with legislation and enforcement, respectively. Only few effects were classified as a clear effect favoring the control (↓), an unclear effect potentially favoring the control (↘), or a null effect (◼). This suggests that across intervention categories, apart from road user behavior/education interventions on their own, interventions targeting RTIs may be effective in reducing crashes, injuries and deaths among pedestrians.

Secondly, *Table 3* underlines that several intervention category-outcome pairs are poorly represented in the evidence base. The intervention categories changes to the road environment and changes to legislation and enforcement had the most complete evidence bases, with identified studies addressing most of the outcomes. However, even for these intervention categories very few or no studies contribute data towards certain outcomes, such as injuries or deaths. For the other intervention categories for which we identified studies, road user behavior/education interventions, combined road user behavior/education and changes to the road environment as well as combined road user behavior/education and changes to legislation and enforcement we found very little evidence.

Lastly, we found the certainty of the evidence, assessed using GRADE, to be low or very low across all intervention category-outcome pairs. These ratings stem primarily from i) the ‘low’ starting point for these bodies of evidence comprising non-randomized study designs, ii) additional risk of bias concerns identified across studies and iii) inconsistency, where effects in both directions (i.e., effects favoring both the intervention and control, regardless of whether clear or unclear) were identified for the same intervention category-outcome pair.

## Discussion

In this systematic review, we assessed the effectiveness of interventions to reduce pedestrian road traffic injuries globally. We identified studies that assessed interventions altering the road environment, legislation and enforcement, road user behavior and education, and combinations of these. Overall, the body of evidence suggests that road environment, legislation and enforcement interventions alone and combined with road user behavior/education interventions may improve pedestrian safety.

Although the highest burden of RTIs is in low- and middle-income countries (LMICs) [42], this review found that, except for two studies from Tanzania [37,40], all the studies were from high income countries. It is possible that either there are few pedestrian intervention studies conducted in LMICs or they are not published. The limited studies in LMICs are a major setback in the identification and implementation of evidence-based interventions to reduce the high burden of pedestrian RTIs. Given the differences in levels of infrastructure development, fleet and road user mix, and road transport resources between LMICs and HICs, interventions that have been proved to be successful in HICs might not be implementable, or successful in LMICs. This points to the urgent need for high-quality studies evaluating such interventions in the specific context of LMICs. As the bulk of pedestrian deaths are in LMICs, and as they constitute an important proportion of all road deaths globally [1], rigorous local evaluation studies must be conducted for a chance to attain global road safety targets, especially to halve the number of road deaths by 2030 [43].

Based on the GRADE certainty of evidence, the identified body of evidence were at best rated as low, and many were rated very low. This is likely a reflection of the difficulties related to feasibility and resources of conducting evaluations of road traffic interventions. By nature, most of the identified interventions require changes to infrastructure and/or an intervention or policy at the population-level; for such studies, study design features such as randomization, blinding and preventing contamination across study groups may be challenging or even impossible. However, several studies did apply study design and analysis methodologies that aimed to identify suitable control sites and limit the influence of potential confounders [26,27,30,34]. Future studies should further consider how to improve the internal validity of such evaluations by focusing on study designs and analysis techniques which help to minimize confounding and other risks of bias.

Interventions were grouped into broad categories, but within each category the interventions varied greatly. For instance, a recommendation to favor infrastructural over behavior change interventions would need to be further clarified since the infrastructural interventions included such diverse actions as construction of speed humps and curb extensions, enhanced road signage, construction of a new motorway, and road closures. Behavior change and education included interventions as varied as safety education in elementary schools and a hospital-based injury prevention program. This heterogeneity might have blunted the distinctions and comparisons between the broad categories.

The review had some limitations. We conducted searches across a broad range of databases, without temporal or geographical restrictions, in aiming to identify all published studies. However, given that studies in this field are published in multiple research fields, such as, health, economics, engineering, urban development, and social sciences, among others, it is possible that some relevant studies were not identified. Although we did not exclude studies from other languages per se, it is possible that we missed relevant studies in languages other than English. Additionally, through our eligibility criteria we may have deemed some studies ineligible that could have provided additional relevant information. For example, we did not consider studies that combined pedestrians with other vulnerable road users such as bicyclists. Although these potentially contained relevant information for RTIs, conclusions specific to pedestrians could not be made. We were unable to conduct meta-analyses due to the methodological and reporting heterogeneity of included studies; we instead relied upon the most up-to-date narrative and graphical summary approach to meaningfully synthesize and summarize findings.

Until there are more studies conducted and rigorously evaluated in low- and middle-income countries, it will be difficult to determine what interventions to recommend with confidence. Cost and pre-existing road infrastructure will be major factors in deciding what interventions can be implemented in the countries with the largest pedestrian injury burdens.

## Conclusion

The systematic review has implications for further studies within the African setting to evaluate the available pedestrian safety interventions for their effectiveness. Additionally, all the road safety stakeholders should be involved in scaling up measures that are proven to be effective in reducing pedestrian crashes.

## Supporting information

S1 Checklist PRISMA_2020_Checklist-RT4 review. (DOCX)

S1 Table MEDLINE search strategy used for this review. (DOCX)

S2 Table Risk of bias detailed summary tables. (DOCX)

S3 Table Sample of excluded studies. (DOCX)

S1 Text Systematic review protocol. (DOCX)
