# Supplementary material for: Interventions to reduce pedestrian road traffic injuries: A systematic review of randomized controlled trials, cluster randomized controlled trials, interrupted time-series, and controlled before-after studies
Source: PLoS One. 2022 Jan 24;17(1):e0262681. doi: 10.1371/journal.pone.0262681 (PMC8786203; doi:10.1371/journal.pone.0262681)
Supplement: S1 Table — (DOCX) [file pone.0262681.s002.docx]

S2 Table. MEDLINE Search strategy used for this review

| Search  component | | Searches | | Results |
| --- | --- | --- | --- | --- |
| Population | 1 | | (pedestrian* or pietons* or vulnerable road user* or VRU*non-motori* or non motorist or non motorists or safety).mp | 960358 |
| Intervention | 2 | | (road* or street* or traffic or vehicle*).ti,ab. | 253914 |
| Outcome | 3 | | (accident* or crash* or collision* or injur* or wound* or mortality or death* or fatal* or hospitalization* or hospitalisation* or hospital admission or hospital admissions).ti,ab | 3006616 |
| PIO combined | 4 | | 1 and 2 and 3 | 11529 |
| Study design | 5 | | randomized controlled trial/ or randomized/ or controlled study/ or comparative study/ or clinical study/ or quasi experimental study/ or experimental study/ or control group/ or follow up/ or prospective study/ or retrospective study/ | 8331430 |
| … | 6 | | (randomized or placebo or (random* and trial* and group*)).mp. | 1191798 |
| … | 7 | | (control group* or follow-up stud* or follow-up assessment or prospectiv* or non-random*or nonrandom*).mp. | 1576998 |
| … | 8 | | (before after stud* or "before and after" or time series or time-series or retrospective* or longitud* or (controlled and cohort* and stud*)).mp. | 1971894 |
| … | 9 | | (controlled before or pre test or pretest or posttest or post test or pre intervention or post intervention).mp. | 54228 |
| … | 10 | | or/5-9 | 9724847 |
| PIOS combined | 11 | | 4 and 10 | 4146 |
