# Supplementary material for: Interventions to reduce pedestrian road traffic injuries: A systematic review of randomized controlled trials, cluster randomized controlled trials, interrupted time-series, and controlled before-after studies
Source: PLoS One. 2022 Jan 24;17(1):e0262681. doi: 10.1371/journal.pone.0262681 (PMC8786203; doi:10.1371/journal.pone.0262681)
Supplement: S2 Table — (DOCX) [file pone.0262681.s003.docx]

**S3 Table. Risk of Bias detailed summary tables**

# Controlled interrupted Time Series (cITS) studies

| **Bias domain** | Noland2007 | Porter2018 | Song2019 |
| --- | --- | --- | --- |
| 1.Were baseline outcome measurements similar? | ***Low Risk:*** This is not reported, however given that this is an ITS analysis, this likely did not lead to biased results; what important is that any differences remain relatively stable over time | ***Low risk:*** The authors report that the baseline parameters were comparable between the study units. However, the authors don't report the baseline outcome measurements regarding pedestrian outcomes. Given that this is an ITS analysis, this likely did not lead to biased results; what important is that any differences remain relatively stable over time | ***Low risk:*** To ensure that the selected control group and the treated group are comparable, the sample odds ratios between the two groups annual crash numbers during the pre-intervention period, were calculated |
| 2.Were baseline characteristics similar? | ***Low risk:*** This is not reported, however given that this is an ITS analysis, this likely did not lead to biased results; what important is that any differences remain relatively stable over time | ***Low risk:*** Baseline characteristics for the comparison group are mentioned in text but no data is presented: "The 13-state South census region comparison group includes states that when compared with Florida have a variety of comparable measures, including (but not limited to) miles of public roads, fatalities per vehicle mile traveled, vehicles per person, percentage of occupied housing units with no vehicle available, age distribution, sex ratios, median household income, and percentage of people whose income is below the poverty level. " | ***Low risk***: “To each treated street section, five control street sections having the smallest diﬀerence in propensity score, comparing with the treated section, were identified as matches" |
| 3. Were incomplete outcome data adequately addressed? | ***Unclear risk:*** This is not reported; it can likely be assumed the routine data surrounding traffic accidents, especially those resulting in deaths and/or severe injuries are well kept. | ***Unclear risk:*** The authors do not report any missing outcome data so bias was unlikely. | ***Low risk:*** The aggregated traffic data set had multiple missing values, thus could not be used in the ITSA as an exposure variable, so number of lanes was used as an exposure in the ITSA instead. Despite the missing values in traffic volume data, averaged traffic volumes over the pre-intervention months could be calculated and were sufficient to be used in the PSM for control group selection" |
| 4. Was knowledge of the allocated interventions adequately prevented during the study? | ***Low risk***: No, but given the very objective nature of the outcome and its measurement, this likely did not influence study results | ***Low risk:*** No blinding, as the objective outcome was analyzed using Florida’s pedestrian fatality counts from January 1975 through December 2013) from the National Highway Traffic Safety Administration Fatality Analysis Reporting System so bias is not likely to be introduced. | ***Low risk***: No blinding, but the objective nature of the outcome means this did not likely lead to meaningful bias. |
| 5. Was the study adequately protected against contamination? | ***High risk***: The control site contains each of the intervention sites assessed, meaning that each is by nature contaminated. Since the authors did not report the absolute number for the control period, it is unclear how severe this contamination is. | ***Low risk:*** The authors explicitly state that the 13 states used in the comparison group had not received the intervention by December 2013. However, it is worth noting that the comparison groups are located in the same census region as the intervention site (Florida) | ***High risk***: Intervention and control sites located in the same region, meaning that contamination was likely influential |
| 6.Was the study free from selective outcome reporting? | ***Low risk:*** No evidence of selective outcome reporting | ***Low risk:*** The authors reported all results and seem not to have concealed any results | ***Low risk:*** Given the data sources used. All outcomes seem to have been reported. |
| 7.Was the study free from other risks of bias? | ***Low risk:*** There is no evidence of other risk of bias. | ***Low risk:*** There is no evidence of other risk of bias | ***Low risk:*** None identified |
| 8. Was the intervention independent of other changes? | ***Low risk:*** The use of an ITS study design and the addition of a control should ensure that the intervention was independent of other changes | ***Low risk:*** The authors do not report any kind of co-interventions during the implementation period of the intervention. | ***Low risk:*** The authors do not report any kind of co-interventions during the implementation period of the intervention. |
| 9. Was the shape of the intervention effect pre-specified? | ***Low risk:*** The point of the intervention is the point of analysis; authors explicitly describe that they expect and test for a step change | ***Low risk:*** Point of intervention is included in the analysis. This is largely due to the nature of the intervention which has limited impact on data collection since it doesn’t require diversion of traffic as is the case with other interventions like road environment interventions | ***Low risk***: Point of intervention is included in the analysis. This is largely due to the nature of the intervention which has limited impact on data collection since it doesn’t require diversion of traffic as is the case with other interventions like road environment interventions |
| 10. Was the intervention unlikely to affect data collection? | ***Low risk:*** Since the study relied on data that was obtained from the STATS19 database (1991–2002) and Transport for London (2003–2004), it is unlikely that the intervention would affect data collection. | ***Low risk:*** The authors used routinely collected data and the intervention couldn’t have affected data collection. | ***Low risk:*** Intervention was unlikely to affect data collection |

# unInterrupted Time Series (uITS) studies

| **Bias domain** | Preusser1982 | Novoa2011 | Kweon2009 | Durkin1999 |
| --- | --- | --- | --- | --- |
| 1.Was the intervention independent of other changes? | ***High risk:*** There was a general rise in the crashes number at all sites where the intervention had been implemented, however this may not necessarily be attributed to the intervention itself as the authors did not control for confounding factors in their analysis. | ***High risk***:  A penalty points system was implemented in 2006 (approximately one year before the intervention); this could have influenced the measured intervention effectiveness. A dummy variable was included for this, but this assumes that there was a clear immediate impact of the intervention, rather than a delayed or gradual impact.   Additionally, there were other road traffic initiatives occurring, and the study authors also mention the financial crisis as potentially having impacted driving practices | ***High risk:*** No comparison states were used although these were available based on their cross-sectional analysis. ITS models did not account for other variables such as traffic volume | ***Low risk:*** There is no evidence showing that there was any major intervention in this area that coincided with the timing of the intervention. |
| 2. Was the shape of the intervention effect pre-specified? | ***High risk***; Authors seem to have chosen any 12 months before and 12 months after the intervention. There is no discussion of the intervention point or whether slope and/or immediate impact should be assessed. | ***Low risk***: The time point of the intervention was used in the analysis as the interruption point | ***Unclear risk:*** Authors used statistical significance for model selection, which also determined how the intervention was modelled. | ***Low risk:*** The point of analysis is the point of intervention; Information for analysis was gathered from the point in time the intervention was implemented |
| 3.Was the intervention unlikely to affect data collection? | ***Low risk:*** Since the authors used routinely collected data, the intervention was unlikely to affect data collection | ***Unclear risk:*** Although the collection system itself did not change; it is conceivable that the reporting practices of law enforcement individuals intensified due to the active traffic campaigns. | ***Low risk:*** FARS data collection probably did not change after the intervention | ***Low risk:***  The study relied on Northern Manhattan Injury Surveillance System Database that included both fatal and severe nonfatal injuries occurring in a population of ;100 000 children <17 years of age and Hospital discharge and death certificate data on severe pediatric injuries…which were linked to census counts to compute incidence. Intervention was not likely to affect data collection. |
| 4.Was knowledge of the allocated interventions adequately prevented during the study? | ***Low risk;*** Knowledge of the intervention could not influence the measure of the outcome. | ***Low risk***: Knowledge of the allocated interventions did not likely lead to bias in the study | ***Low risk:*** Objective outcome measure | ***Low risk:*** The researchers were not blinded as the study relied on Northern Manhattan Injury Surveillance System Database and Hospital discharge and death certificate data therefore the outcome could not be influenced |
| 5.Were incomplete outcome data adequately addressed? | ***Low risk:*** *"To arrive at a realistic estimate of the percentage of all pedestrian accidents involving a right-turning motor vehicle at a signalized location, the unknowns were distributed proportionately into the known categories."* The analysis was done using periods before and after in which data is available | ***Low risk:*** This is not reported; however, it is likely that the routine data collection system in Spain is largely complete. | ***Unclear risk***: Unclear if missing data was a problem for this study | ***Low risk:*** Data was similar for both the pre-intervention period and intervention period. |
| 6. Was the study free from selective outcome reporting? | ***Low risk:*** The authors report all the outcomes they set out to report in the objective, studying the effect of RTOR on pedestrian and motorcyclists. | ***Low risk:*** No evidence of selective outcome reporting | ***High risk***: Because statistical significance was used for model definition, coefficients for the intervention variable were available for all models. Different levels of statistical significance were used across analyses. | ***Low risk:*** No evidence of selective outcome reporting. The study set out to describe the incidence of severe traffic injuries before and after implementation of a comprehensive, hospital-initiated injury prevention program aimed at the prevention of traffic injuries to school-aged children in an urban community. |
| 7. Was the study free from other risks of bias? | ***Low risk:*** No other bias evident | ***Low risk***: No other sources of bias observed | ***Low risk:*** No other sources of bias. | ***Low risk:*** No other risks of bias identified |

# Controlled Before and After (CBA) studies

| **Bias domain** | Zimmerman2015 | Yannis2017 | Yamanaka1998 | Steinbach2010 | Rothman2017 | Poswayo2018 |
| --- | --- | --- | --- | --- | --- | --- |
| 1.Were baseline outcome measurements similar? | ***High risk:***  Although the authors report similarity between study groups, the numbers of RTIs at baseline differ… | ***Low risk:*** The control sites were selected to be similar to the treatment site. | ***Low risk:*** The authors measured baseline outcomes for the intervention sites and the control sites; Table 2 shows that, although there is some variation cross sites, levels are general similar. | ***High risk:*** The baseline outcomes were not evaluated for similarity | ***High risk:*** The baseline outcomes were not evaluated for similarity | ***Low risk:*** The authors measured baseline a similar number of injuries at intervention and control sites prior to the intervention: "Data were collected on 12 957 school-aged children in the baseline period. At baseline, 181 RTI were reported (92 control, 89 intervention) to have occurred in the past 12months…" |
| 2. Were baseline characteristics similar? | ***High risk:***  Most of the baseline characteristics were similar but the nature of the road was different with intervention community road being paved by the follow-up time while that in the control community was not paved by follow-up time & Seasonality in road use may have affected the results, because the collection periods were not equivalent and may have been biased by seasonal variation in travel behavior and climate conditions. | ***Low risk:*** The comparison group includes the Municipalities of Holargos and Agia Paraskevi. These Municipalities are located near the study area and as it is shown in Table 2; they all present similar road network, land use and traffic volumes characteristics | ***High risk***: The study reports baseline characteristics for the Intervention areas only reported see ***Table1*** | ***High risk:*** There is no reporting of the baseline characteristics. | ***High risk:*** No consideration of baseline characteristics. | ***Low risk:*** The authors report baseline characteristics for the Intervention and control group: "Schools were divided into intervention and control groups, which were subsequently matched for environmental characteristics and pupil enrolment numbers…” |
| 3.Were incomplete outcome data adequately addressed? | ***High risk:*** Though the study team attempted to reach every household within 200 m of the road, the rural  characteristics of the sites may have prevented the study team from capturing every household | ***Low risk:*** In addition, crash data were available for the entire period both before and after the implementation of these measures. And the authors used routinely collected data | ***Unclear risk***: The authors do not discuss any missing data. Given that the accident data analyzed included only injury accidents based on a map made by the police agency in each ward of Osaka city. Therefore, it unlikely that missing data meaningfully biased results for the two study periods. | ***Unclear risk:***  No mention of the completeness of the data that is used. | ***Low risk:*** The authors excluded sites with incomplete or insufficient data from their analysis | ***Low risk;*** The authors do not report any missing out come data. Given that the study had a robust study design, the collection of primary data was essential to accurately capture the impact of RTI. There bias was less likely. |
| 4. Was knowledge of the allocated interventions adequately prevented during the study? | ***High risk***: The nature of the intervention could not allow for concealment of knowledge of the allocated arm; outcomes were self-reported, thus some sensitization may have been likely | ***Low risk:*** Objective outcomes calculated from routine data assessed, likely no bias | ***Low risk:*** The outcome measure was objective and therefore this risk of bias was minimized | ***Low risk:*** Knowledge of the intervention does not influence the reporting and recording of causalities by the police who were the source of the data that was used. | ***Low risk:*** Knowledge of the intervention does not affect the outcome measure. | ***High risk:*** The deployed research assistants that gathered the primary outcome data were blinded; however, the collected outcomes were self-reported, meaning that the intervention itself could have caused some sensitization (e.g. differential recall) among participants at intervention schools. |
| 5. Was the study adequately protected against contamination? | ***High risk:*** Given the fact that part of the intervention involved sensitization and provision of reflectors, it is likely from control sites gained knowledge of the intervention. | ***Low risk:*** The intervention was road environment change which was not carried out in the neighbouring control sites. | ***Unclear risk:***  The intervention and control are found in the same geographical area that is the Osaka City. It is unclear whether the proximity of these sites could lead to bias. | ***High risk:***  Low speeds in the 20mph zones can have an effect on zones that surround them as the drivers try to compensate for any delays experienced in the 20mph zones. | ***High risk***: The study sites were selected to be geographically separated by a minimum distance of 30 meters. | ***Low risk:*** Geographical non-adjacency considered in sight selection to reduce risk of contamination; some still possible, but unlikely to meaningfully bias results. |
| 6. Was the study free from selective outcome reporting? | ***Low risk***: The study adequately reports as set out in the objectives. | ***Low risk:*** The results of the study adequately meet the objectives set out. | ***Low risk:***  Analyses were conducted as defined for different variables and effects from these analyses are reported in the results. | ***Low risk***: The study extensively reports the outcome that they set out to investigate in the objectives. | **Low risk:** The authors clearly report all the outcomes set out in the objectives of the study. | ***Low risk:*** The study was robust in nature and all the main outcome variables are thoroughly reported in the result sections. |
| 7.Was the study free from other risks of bias? | ***Low risk:*** The study relied on self reported data from household members. This introduces a high potential for bias due to potential for incomplete outcome data reporting and the authors also report that some households may not have been reached by the research team especially in rural areas "The intervention community may have also experienced RTI sensitization as a result of the road safety interventions and may have been more likely to recall and report RTIs" | ***Low risk:*** No other risks of bias identified | ***High risk:***  This study considers only injured accidents, but there are quite frequent accidents without injury in Japan, due to low vehicle speed along narrow streets. | ***Low risk:*** No other risks of bias identified | ***Low risk:*** No other risks of bias identified | ***High risk***:  The authors discuss some limitations to the study which may have introduced bias and included: "When inquiring about RTI, it is not specified if the RTI took place within the school district, where the benefit of infrastructure enhancements would have been seen.“ |

# CBA studies continued

| **Bias domain** | Polus 1978 | Persaud1997 | Olsen2016 | Naznin2016 | Kloeden2006 | Green2014 |
| --- | --- | --- | --- | --- | --- | --- |
| 1.Were baseline outcome measurements similar? | ***High risk:*** Not clearly reported  . | ***Unclear risk:***  Pre-intervention crash data for control sites are not provided; it is unclear, however, given the type of analysis applied here, that differences here would bias the results meaningfully. | ***High risk:***  Outcome measures were different across sites at baseline | ***Low risk***: Differences at baseline factored into the analysis | ***High risk:*** The authors did not perform any baseline assessments | ***Low risk:***  Although there were slight differences in outcomes, the analysis applied in this study should account for these. |
| 2. Were baseline characteristics similar? | ***High risk***:  Not assessed | ***Unclear risk***: No characteristics provided, however the empirical Bayes analysis likely addressed differences. | ***High risk:***  Not assessed | ***Low risk:***  Comparison sites were chosen along the same tram route as the treatment sites in order to account for temporal, operational and regional factors so bias was unlikely to be introduced. | ***High risk:*** The characteristics were not examined. | ***Low risk:*** No data on baseline differences between groups were presented, however these should have been accounted for through the analysis. |
| 3.Were incomplete outcome data adequately addressed? | ***Unclear risk***: No signs of incomplete outcome data since they used routinely collected data | ***High risk:***  Authors discuss two main data limitations and the extent to which they affect the results is unclear. "**First**, there was the unexplained year-to-year variation in crash counts for the comparison group. **Second**, traffic volume estimates, though carefully derived, are only as good as the raw data on which they are based. The sparsity of raw data, and several apparent inconsistencies therein, placed some limitations on the analysis, the main one being the inability to account for year-to-year variation in traffic volumes." Therefore, these were like to influence the outcome. | ***Low risk:*** Authors discuss potential limitations of missing or under-reported data in the discussion; however, these are likely relatively high quality | ***Unclear risk:*** Authors do not discuss missing data. Given that Crash data were extracted from the public version of Crash Stats, a crash recording system developed and populated by VicRoads and Victoria Police. | ***Low risk:*** No evidence of missing data | ***Low risk:*** We have the opportunity to exploit relatively robust data sets (for instance, more detailed travel diary data than available for the rest of the country, and more complete road traffic injury data) |
| 4. Was knowledge of the allocated interventions adequately prevented during the study? | ***High risk***: No blinding; given, however, that hard objective outcomes were assessed, this likely had no influence on the study results | ***Low risk***: Knowledge of allocation was not prevented; however, given the nature of the intervention and the objectivity of the data, it is unlikely that this meaningfully influenced results. | ***Low risk:*** No blinding; given, however, that hard objective outcomes were assessed, this likely had no influence on the study results | ***Low risk***: Outcomes were assessed from crash data that were extracted from the public version of CrashStats, a crash recording system developed and populated by VicRoads and Victoria Police, likely no bias arising from this objective outcome. | ***Low risk:*** Objective outcomes calculated from routine police data assessed, likely no bias | ***Low risk:*** The outcome variable was not assessed blindly; the researchers used a routine data source: STATS19 Road Accident data set (2001– 2009) therefore, this objective outcome was likely unbiased. |
| 5. Was the study adequately protected against contamination? | ***Low risk*** | ***Low risk:***  Given the proximity of intervention and control sites, it is possible that some contamination occurred. This would have resulted in a bias towards the null, however, which would have showed more of an effect. | ***Low risk:*** Possible that changes in the local road structure influenced driving on a broader geographical scale - however this is not likely a serious concern | ***High risk:***  Comparison sites were chosen along the same tram route as the treatment sites; this could have led to contamination at control sites. | ***Unclear risk***: Unclear how close the intervention and control sites were geographically, and whether this could have led to contamination. | ***Low risk:*** The Control Group consisted of adults aged 25 - 59 years who were not affected by the intervention. Some contamination possible, if increased bus riding in young adults bled over to their parents or other adults, however this is unlikely to lead to substantial bias. |
| 6. Was the study free from selective outcome reporting? | ***Low risk*** | ***Low risk:***  No evidence of selective outcome reporting. | ***Low risk:*** No evidence of selective outcome reporting in fact the reporting on the methods and results is very comprehensive | ***Low risk:*** As no study protocol is referenced, this cannot be rigorously assessed. However, a modified analysis was done to adjust for under estimation of the safety impacts of the platform tram stops as a result of large differences in passenger stop use between the treatment and control sites, suggesting differences in crash risk exposure. | ***Unclear risk***: No evidence of selective outcome reporting. | ***Low risk***: The study was free from selective outcome reporting as all the relevant outcomes were reported in the results sections. |
| 7.Was the study free from other risks of bias? | ***Low risk:***  There is no evidence of other risk of bias. | ***Low risk:***  There is no evidence of other risk of bias. | ***High risk:*** From discussion:  "We have described factors in different areas, such as recent investment in housing, cycle networks and bus lanes in the North area. There are a large number of other factors that contribute to RTAs, such as alcohol and changes to street lighting"  These changes across sites in the city likely introduced bias into the results | ***Low risk:*** There is no evidence of other risk of bias. | ***Low risk***: There is no evidence of other risk of bias. | ***Low risk:*** There is no evidence of other risk of bias. |

# CBA studies continued

| **Bias domain** | Feldman2010 | Ewing2013 | Dimaggio2013 | Choi2013 | Chen2014 | Chen 2013 | Agent1996 |
| --- | --- | --- | --- | --- | --- | --- | --- |
| 1.Were baseline outcome measurements similar? | ***Low risk***:  Treatment sites were matched to the control sites. | ***Low risk:*** Figure 5 shows that outcome levels were slightly different, however the analysis should have addressed this | ***High risk:***  There was no comparison of baseline outcome measures conducted. And treatment sites were selected as having high injury rates hence this may introduce wide differences between the control and treatment sites. | ***Low risk:***  Not a serious risk of bias since a DID analysis was conducted. | ***Unclear risk:***  Some fairly large differences pre-intervention between intervention and control sites; however, authors aim to account for these baseline differences by including them as covariables in the model, and by analysing outcomes as pre- and post-intervention changes | ***Low risk:***  The authors ensured that treated sites and control sites had very similar characteristics in terms of traffic volumes, direction. | ***High risk:***  There is no comparison of baseline outcomes and the authors do not account for baseline differences in their analysis. |
| 2. Were baseline characteristics similar? | ***Low risk:***  Characteristics for the treatment and control sites were selected to be similar. | ***Low risk:***  Baseline characteristics were described for both the comparison and treatment group and were similar for both groups | ***High risk:***  Although its not reported that there were or there was no significant baseline difference, it is noted that implementation of interventions was based on which communities had higher burden of crashes/injuries. The intervention communities could have had inherent differences that placed them at higher risk. | ***Low risk:***  Control group was closely matched to treatment group and the areas designated as a silver zone in earlier years were an appropriate control for the areas designated as such in the later years | ***Low risk:***  Controls were selected using a matching process based on several important characteristics. Table 3 shows that most characteristics were fairly well balanced between intervention and control sites for the four different countermeasure types | ***Low risk:***  “For each of the 13 countermeasures, we also explicitly considered characteristics of the surrounding built environment by selecting a set of comparison sites whose geographical distribution matches those of the countermeasure.” | ***High risk:***  There is no clear mention of the baseline characteristics |
| 3.Were incomplete outcome data adequately addressed? | ***Low risk***: The study selected sites for which data for before and after periods was adequately available | ***High risk****; It was noted that because of a*bsence of consistent traffic volume data for New York City streets, it was impossible to say whether traffic volumes declined after speed tables were installed, an effect that has been well documented for speed humps but appears to be less prevalent with speed tables | ***Unclear risk:***  Authors do not discuss missing data. Given that routine police accident data are used, it is unlikely that missing data meaningfully biased results for the 5 year study period. | ***Low risk:***  The study used Elderly Pedestrian Vehicular Collision data from the Korea Road Traffic Authority (KoRoad) from 2010 to 2015 for both the before and after periods so missing data was unlikely to bias the outcome. This data is routinely collected | ***Unclear risk:***  Authors do not discuss missing data. Given that routine police accident data are used, it is unlikely that missing data meaningfully biased results for the 7 year study period. However, there is a possibility of some data missing since the actual year in which the interventions are set up are excluded from the analysis | ***Low risk:***  Data was systematically collected by the state department and analysis was done based on availability of adequate data points. | ***Low risk:*** There is low risk of incomplete data since data was from the ADD crash data systems collected for state routes |
| 4. Was knowledge of the allocated interventions adequately prevented during the study? | ***Low risk:***  Knowledge of the intervention does not impede the measure of this objective outcome | ***Low risk:***  Objective outcome, thus the lack of blinding should not lead to substantial bias. | ***Low risk***:  Objective outcomes calculated from routine police data assessed, likely no bias | ***Low risk:***  Data on collisions was obtained from Korean Traffic Authority that is routinely collected and unlikely to bias results | ***Low risk:***  Knowledge was not prevented, however this likely did not lead to bias in this study, where a hard outcome, the occurrence of a crash was assessed | ***Low risk:***  The outcome measure which is pedestrian crashes is objective and measure is not affected by knowledge of the intervention. | ***Low risk:***  Knowledge of the outcome could not influence the outcome measured. |
| 5. Was the study adequately protected against contamination? | ***Low risk:***  Given the geographical proximity of intervention and control sites, it is likely that the intervention also influenced driving at the control intersections - however, this would have led to an underestimation of the effect. | ***Low risk:***  The study and control sites were far apart hence a low risk of contamination "By design, the comparison streets were (a) not adjacent to the treated streets so that they were unlikely to get spill over traffic from the treatment and (b) not treated themselves with any of the measures in the New York City toolbox" | ***High risk:***  Therefore, the observed overall decline in school aged pedestrian injury during the study period cannot so be attributed to the SRTS interventions at the relatively small number of 124 schools; the decline is due in part to the improvement of the overall traffic safety environment brought about by other concurrent programs. | ***Low risk:*** Areas designated as silver zones in the earlier years were controls for areas designated as such in the later years and this unlikely to bias | ***High risk:***  Given that all sites are in the same city, it is possible that changes at intervention intersections influenced either behaviour of drivers or pedestrians or the traffic flow at control sites | ***Low risk:***  “To avoid spill over effects—the safety effects on untreated locations simply due to their proximity to the treated locations (Council et al., 2005; Ewing and Brown, 2009), locations that are adjacent to treatment locations were avoided” | ***High risk:***  It might not have been possible to stop the interventions from spilling over the neighbouring counties |
| 6. Was the study free from selective outcome reporting? | ***Low risk:***  The authors adequately report as they set out in the objectives. | ***Unclear risk:*** The fact that there is no description of the data sources makes bias due to selective outcome reporting challenging. | ***Low risk:***  The authors adequately report the results. | ***Low risk:***  The researchers set out to determine the effectiveness of silver zones in reducing elderly pedestrian-vehicle collisions of which they reported on. | ***Low risk:***  No suggestion of selective outcome reporting | ***Low risk:***  The study reported results for all the hypothesis that were stated in the introduction. | ***Low risk:***  The authors report on all the outcomes they set out to report in the study objectives. |
| 7.Was the study free from other risks of bias? | ***Unclear risk:***  Co-interventions happening during the study period (other traffic measures, changes in vehicle flows, etc.) may have biased this study. However, it is unclear whether these would have differentially affected intervention and control intersections, given their proximity to one another. | ***High risk:***  The evaluation did not control for exposure as noted by the authors | ***Low risk:***  No other bias identified | ***Unclear risk:***  The intervention was not independent of other changes as the author mentions of other ten physical elements in the zones which would affect the frequency of collisions. [Traffic Lights, Cross walks, Intersections] | ***Low risk:***  No other bias identified | ***High risk:***  If the “group” variable is severely correlated with the “pretest” variable (crashes in the pre-treatment period), the estimated ˛ (the key mechanism in correcting the RTM problem) may be biased. | ***Low risk***: No other risks of bias assessed. |
