# Supplementary material for: Interventions to reduce pedestrian road traffic injuries: A systematic review of randomized controlled trials, cluster randomized controlled trials, interrupted time-series, and controlled before-after studies
Source: PLoS One. 2022 Jan 24;17(1):e0262681. doi: 10.1371/journal.pone.0262681 (PMC8786203; doi:10.1371/journal.pone.0262681)
Supplement: S3 Table — (DOCX) [file pone.0262681.s004.docx]

**S4 Table. Sample of excluded studies**

| Study ID | Title | Reason for exclusion |
| --- | --- | --- |
| Lama 2007 | Effectiveness of the 2000 - 2006 national road traffic safety program implementation in latvia | Study design: This was a before after study with no control group |
| Hussein2017 | Surface condition and safety at signalized intersections | Outcomes: did not report pedestrian specific outcomes |
| De Pauw & Brijs2014 | To brake or to accelerate? Safety effects of combined speed and red-light cameras | Study design: lacked a comprehensive analysis of the before and after periods |
| Liu 2011 | Effects of transverse rumble strips on safety of pedestrian crosswalks on rural roads in China | Outcomes: combined results across population groups - no specific pedestrian outcomes |
| Lindqvist 2001 | Evaluation of inter-organizational traffic injury prevention in a WHO safe community | Outcomes: lumped included traffic and non-traffic pedestrian injuries together |
| Lienau 1996 | Safety effects of barrier curb on high-speed suburban multilane highways | Outcomes: no pedestrian specific outcomes |
| Li 2018 | Have pedestrian subsystem tests improved passenger car front shape? | Study design: no valid control |
| Leden 2002 | Pedestrian risk decrease with pedestrian flow. A case study based on data from signalized intersections in Hamilton, Ontario | Study design: cross-sectional |
| Le 2017 | Safety evaluation of multiple strategies at stop-controlled intersections | Outcomes: no pedestrian specific outcomes) |
| Kudryavtsev 2012 | Explaining reduction of pedestrian-motor vehicle crashes in Arkhangelsk, Russia, in 2005-2010 | Study design: focus was on overall trends with no pre-post intervention comparison) |
| Macmillan2018 | Controlled before-after intervention study of suburb-wide street changes to increase walking and cycling: Te Ara Mua-Future Streets study design | Study design: study protocol |
| Kirley 2008 | Does the Maryland graduated driver licensing law affect both 16-year-old drivers and those who share the road with them? | Outcomes: no pedestrian specific outcomes |
| Zhang2010 | Evaluation of a School-Based Intervention to Reduce Traffic-Related Injuries among Adolescents in Beijing | Study design: a pre-post test method |
| Weijermar2011 | Ten Years of Sustainable Safety in the Netherlands An Assessment | Study design: time series study without 3 or more time points before and after the intervention |
| VanDerHostA | Changes in crash risk following re-timing of traffic signal change intervals | Study design: no valid control |
