# Supplementary material for: Interventions to reduce pedestrian road traffic injuries: A systematic review of randomized controlled trials, cluster randomized controlled trials, interrupted time-series, and controlled before-after studies
Source: PLoS One. 2022 Jan 24;17(1):e0262681. doi: 10.1371/journal.pone.0262681 (PMC8786203; doi:10.1371/journal.pone.0262681)
Supplement: S1 Text — (DOCX) [file pone.0262681.s005.docx]

**S5 Text. Systematic review protocol**

**Interventions to prevent road traffic injuries and deaths among pedestrians: a protocol for a global systematic review**

**Authors:** Stellah Namatovu, Bonny Enock Balugaba, Kennedy Muni, Albert Ningwa, Linda Nsabagwa, Fredrick Oporia, Arthur Kiconco, Patrick Kyamanywa, Milton Mutto, Jimmy Osuret, Eva A. Rehfuess, Jacob Burns, Olive Kobusingye

Background

### **Description of the condition/issue**

Road traffic injuries are a substantial burden across all regions of the world and the 8^th^ leading cause of the global burden of disease.^1^ Globally for both sexes, road traffic injuries were the third leading cause of disease burden in the age group 15-55 years, preceded only by HIV/AIDS and unipolar depression.^2^ Between 1990 and 2010, the fraction of global deaths due to injuries (5·1 million deaths) rose from 8.8% to 9.6% , a change largely driven by a 46% rise in deaths from road traffic crashes and falls ^1^.

However, the road traffic burden is greatest in low and middle-income countries (LMICs) in the African, Eastern Mediterranean, and Western Pacific regions.^2^ Although the African region remains the least motorized of the six World Health Organization (WHO) regions, it suffers the highest rates of road traffic fatalities, with 37 of 44 surveyed countries in 2010 having death rates well above the global average of 18.0 deaths per 100 000 population ^3^.

Over a third of road traffic deaths in LMICs are among pedestrians and cyclists ^3^, yet less than 35% of LMICs have policies in place to protect these vulnerable road users. While 22% of the global road traffic deaths are among pedestrians, that proportion is 38% in the African region. This number is likely an underestimate of the true pedestrian burden, as minor and moderate pedestrian injuries probably go unreported.^4^

### **Description of the Intervention**

Although the global risk for road traffic crashes is broadly known and several interventions to mitigate the burden among pedestrians have been widely implemented, the effectiveness of these interventions and the capacity for their implementation vary across regions and countries. These interventions can be categorized based on the aspect they address, and fall in the broad categories of road environment, legislation and enforcement, road user behavior, and vehicle design and condition.

### **How the intervention might work**

The Haddon Matrix is a framework that aids the identification of interventions to prevent injury, and is relevant for understanding how interventions may work to reduce the burden from road traffic crashes on pedestrians.^5,6^ The framework incorporates various components, including the population at risk of injury, the environment/context in which the injury takes place, the agent (cause of the injury), and the injury issue to be addressed.^7^ Potential interventions are organized by when they are expected to prevent or mitigate injury, i.e. before the event (primary prevention), during the event (secondary prevention), and post-event (tertiary prevention). Some interventions may fall under multiple categories (e.g. traffic calming interventions). Table 1 provides an example of how the Haddon Matrix can be applied for identification of potential interventions to reduce road traffic injuries and fatalities among pedestrians ^8^. For example, traffic calming features such as speed humps and rumble strips are pre-event interventions targeting the road environment in order to enhance pedestrian safety. On the other hand, vehicle standards for frontal features are an intervention targeting the agent (car) in order to minimize pedestrian injury during an event (a crash).

### Table 1: An example of the Haddon Matrix applied to pedestrian-impacting interventions

|  | | **Host (pedestrian)** | **Agent (vehicle)** | **Physical & social environment** |
| --- | --- | --- | --- | --- |
| **Pre-event**  **(pre-crash)** | Crash prevention | - Pedestrian visibility enhancement (e.g. bright clothes)^b^ - Education and behavioral interventions regarding road safety, e.g. on dangers of mobile phone use and alcohol use while on the road)^b^ | - Vehicle inspection and roadworthiness^a^ - Vehicle maintenance (e.g. braking)^c^ - Automatic emergency braking^c^ | - Traffic calming features (e.g. speed humps, traffic lights, and rumble strips)^d^ - Speed limits^d^ - Speed zones^d^ - Speed cameras^d^ - Street lighting & visibility enhancement^d^ - Separation of vulnerable road users from fast motorized traffic^d^ - Pedestrian safety facilities and walkways^d^ - City-wide measures to reduce pedestrian interaction with roads carrying fast motorized traffic through land-use planning^d^ |
| **Event**  **(crash)** | Injury prevention during crash |  | - Vehicle standards for frontal features protective of pedestrians^c^ | - Traffic calming features (e.g. speed humps and rumble strips)^d^ |
| **Post-event**  **(post-crash)** | Mitigating any effects of the crash |  |  | - Emergency medical services (pre-hospital care system) - Good Samaritan laws^a^ - First responders/lay responders |

a-Legislation and enforcement; b- road user behavior; c-vehicle design and condition; d-road environment

### **Why is it important to do this review?**

There is increasing pressure for low income countries such as Uganda and Rwanda, to invest in interventions to reduce pedestrian injuries and deaths. Most high income countries (HICs) have reduced road deaths over the last few decades, but their road environments, population structures, and transport systems are markedly different from those of low income countries (LICs) where the burden is currently the highest. There is little evidence to date on intervention effectiveness, thus little help for LICs with regard to prioritizing and decision-making. Moreover, previous systematic reviews on pedestrian safety either focused on interventions in HICs, or on a narrow range of pedestrian interventions.^4^ This review will include all types of pedestrian interventions regardless of where they were implemented.

# Objectives

We aim to assess the effectiveness of interventions to reduce pedestrian injuries and deaths.

# Methods

Throughout the review we will apply pre-defined, systematic, and standardized review methods based on guidance published by Cochrane.^9^

## Criteria for considering studies for this review

### *Types of studies*

Based on our experience in this research field as well as several published reviews on similar topics, we expect to identify little or no evidence applying randomization to assess intervention effectiveness.^4,10-12^ We will, therefore, also include certain non-randomized study designs, which have ensured a certain level of internal validity through specific study design features. These study design features include the consideration of underlying trends through the assessment of multiple time points before and after the intervention, and/or the consideration of concurrent trends on a wider geographical scale through the inclusion of one or multiple control sites.

We will thus include the following study designs:

- **Randomized controlled trials (RCTs)**: Experimental studies in which study subjects or units are randomly allocated to intervention or control group.
- **Cluster randomized controlled trials (c-RCTs)**: Experimental studies in which clusters (e.g. study sites, road environments such as junctions or pedestrian crossings) are allocated to intervention or control using methods of randomization.
- **Interrupted time-series (ITS) studies:** Studies that use observations at multiple time points before and after an intervention. The design attempts to detect whether the intervention has had an effect greater than any underlying trend over time. ITS studies can be either uncontrolled (u-ITS) or controlled (c-ITS); as consistent with guidance published by the Cochrane Effectiveness of Practice and Organization of Care (EPOC) Group, ITS studies must have at least three data points before and after a clearly defined intervention (in terms of content and timing).^13^
- **Controlled before-after (CBA) studies**: Studies in which observations are made before and after the implementation of an intervention, both in a group that receives the intervention and in a control group that does not. Cochrane EPOC recommends only the inclusion of studies where at least two intervention and two control sites were assessed.^13^ We will, however, only require studies to have at least one intervention and one control site, as we expect that such studies may provide valuable evidence for this review. Additionally, as per the newly developed ROBINS-I risk of bias tool, we consider the number of sites in CBA studies as a risk of bias issue, not as an inclusion criterion.^14^

We will include original research studies published in any language regardless of their publication status and with no restriction on the year of publication. Modeling, simulation and qualitative studies will be excluded. However, qualitative findings that are part of the included intervention studies will be incorporated, where possible, to provide context to the study’s findings.

### *Types of populations and setting*

We will include studies assessing the effects of interventions targeting pedestrians of any age. We will define pedestrians as per the World Health Organization, which defines a pedestrian as “any person who is travelling by walking for at least part of his or her journey. In addition to the ordinary form of walking, a pedestrian may be using various modifications and aids to walking such as wheelchairs, motorized scooters, walkers, canes, skateboards, and roller blades”.^15^ Persons “running, jogging, hiking, or sitting or lying down in a roadway” are also considered pedestrians.^15^ We will exclude persons using other modes of transportation (e.g. automobile drivers and passengers, public transport riders, motorcyclists, bicyclists, etc.). Mixed-population studies, where both pedestrians and non-pedestrians are included, will only be included if effect estimates are provided separately for pedestrians, or can be calculated from the study data.

We will include pedestrian interventions implemented in environments of all resource levels.

### *Types of interventions and comparison*

We will include interventions belonging to four distinct categories, i.e. road environment, legislation and enforcement, the vehicle design and condition, and road user behavior; these four categories will provide the basis for synthesizing and interpreting findings. Although these categories do not directly reflect the terminology of the Haddon Matrix, they are conceptually consistent (See Table 1) and will allow us to clearly structure the review.

Some examples of each of these types of interventions are as follows:

- Road environment: separation of vulnerable road users from fast motorized traffic,
- Legislation and enforcement: alcohol laws, mobile phone use laws,
- Vehicle design and condition: frontal features protective of pedestrians,
- Road user behavior: pedestrian visibility enhancement (e.g. bright clothes), and curricula teaching school children how to identify safer routes to school.

We will include both those interventions directly targeting pedestrians and those not directly targeting pedestrians but expected to have an indirect effect on them. For example, speed limits target drivers but they have an indirect effect on the risk and severity of pedestrian injury. The review will not cover post-crash interventions (e.g. pre-hospital emergency and rehabilitative services) although these are included in Table 1 to illustrate the whole Haddon Matrix.

We expect most comparisons to be no intervention or ‘business as usual’, however given the expected heterogeneity of included studies, we will not exclude studies based on the comparison.

### *Types of outcome measures*

#### Primary outcomes

We will include all studies reporting data on at least one of the following primary outcomes:

- Pedestrian crashes: a measure of the number, proportion or rate of crashes in which pedestrians were involved.
- Pedestrian injuries: a measure of the number, proportion or rate of pedestrian injuries.
- Pedestrian hospitalizations: a measure of the number, proportion or rate of pedestrians hospitalized with road traffic-related injuries.
- Pedestrian deaths: a measure of the number, proportion or rate of pedestrian deaths

Secondary outcomes

In studies including at least one primary outcome, we will also focus on the following secondary outcomes:

- Cost of pedestrian crashes and injuries
- Number or proportion of pedestrian disabilities
- Number of arrests for drunk driving
- Number of speeding tickets
- Change in pedestrian knowledge (for educational interventions)

Adverse outcomes

We will extract data on any adverse effects from the interventions included in the review. Such adverse effects might include displacement of accidents and/or a traffic volume to other parts/surrounding road networks, and traffic congestion at intersections.

## Search methods for identification of studies

### *Electronic searches*

We will perform searches in the following electronic databases:

- MEDLINE
- EMBASE
- Web of Science
- WHO Global Health Index
- Health Evidence
- Transport Research International Documentation (TRID)
- ClinicalTrials.gov

Our search strategy will be based on “population”, “intervention”, “outcome” and “study design” search blocks. The Ovid strategy to be applied for MEDLINE and EMBASE, and adapted to all other databases, is provided in *Appendix 1*. The search strategy was developed iteratively by the review team, with each version being tested against a set of test studies eligible for inclusion and an initial screening of 100 titles and abstracts.

### *Searching other resources*

In addition to the electronic databases, we will search previously published related reviews, of which we are already aware, as well as any reviews we identify during the study selection process, search the reference lists of included studies, and consult a group of decision-makers and researchers in the injury field to ask whether we have missed any important studies.^4,10-12^

## Data collection and analysis

### *Selection of studies*

Two reviewers will screen all titles and abstracts independently to determine eligibility against the inclusion criteria. Any disagreement between the two reviewers will be discussed and resolved by consensus. For all studies deemed potentially eligible or unclear at the title and abstract screening stage, we will retrieve the full texts, and these will be assessed for eligibility, analogous to the process described above. At this stage, all studies not meeting the eligibility criteria will be excluded, and we will document all reasons for exclusion. Disagreements between the two reviewers regarding eligibility at any stage will be resolved through discussion with a third reviewer, if necessary. In order to aid in the title/abstract and full text screening, we developed a title/abstract screening guidance and a full text eligibility assessment form (see Appendices 2 and 3). Additionally, we will conduct an initial calibration exercise for both title/abstract and full text screening, to ensure that we all screen in a standardized manner.

We will use EndNote software to manage retrieved studies and to remove duplicate reports of the same study and (https://rayyan.qcri.org/) to manage the title and abstract screening process.

### *Data extraction and management*

Two reviewers will independently extract data from all included studies using a standardized data extraction form. We will pilot the data extraction form across the review team to ensure information is captured in a standard manner. Any discrepancies will be resolved through discussion, and by consulting a third reviewer, if necessary.

We will extract the following data:

- Publication details: author name, journal, year of publication.
- Study design and methods: sampling strategy, sample size and number of sites, response rate, randomization of clusters or individuals, number and selection of intervention and control groups and/or sites.
- Participants: PROGRESS-Plus characteristics that can be used to identify disadvantaged groups and that allow us to differentiate the effects of the intervention across social categories (place of residence, race/ethnicity, occupation, gender/sex, and age).^16^
- Intervention: description of the intervention theory, intervention design (components and timing of execution), and intervention delivery (delivery agent and setting), level of implementation.
- Comparison: description of the comparison against which the intervention would be compared.
- Statistical analysis: statistical testing, model selection, variable selection, and other important aspects regarding the analysis.
- Outcome measures and effect estimates: relevant effect estimates, including the RR, MD, where reported, for all primary and secondary outcomes listed above.
- Setting and context: country of intervention and urban vs. rural area.
- Funding, sponsorship, conflicts of interest: all details on study and/or intervention funders and sponsors, as well as any information regarding declarations or conflicts of interest.

### *Assessment of risk of bias in included studies*

The Cochrane ‘Risk of bias’ tool, as modiﬁed by Cochrane EPOC, is widely used and validated for systematic reviews including a wide range of study designs. Due to the different sources of bias common to the different study designs, separate criteria are applied to controlled studies (RCTs, c-RCTs, CBAs and c-ITS) and for u-ITS.

For controlled studies, the assessment is based on the following aspects:

- Was the allocation sequence adequately generated?
- Was the allocation adequately concealed?
- Were baseline outcome measurements similar?
- Were baseline characteristics similar?
- Were incomplete outcome data adequately addressed?
- Was the knowledge of the allocated intervention adequately prevented during the study?
- Was the study adequately protected against contamination?
- Was the study free from selective outcome reporting?
- Was the study free from other risks of bias?

For u-ITS, the assessment is based on the following aspects:

- Was the intervention independent of other changes?
- Was the shape of the intervention effect pre-specified?
- Was the intervention unlikely to affect data collection?
- Was knowledge of the allocated interventions adequately prevented during the study?
- Were incomplete outcome data adequately addressed?
- Was the study free from selective outcome reporting?
- Was the study free from other risks of bias?

Using these criteria, two authors will assess the risk of bias of included studies independently and in duplicate. Any disagreements will be resolved through discussion, and by involving a third author, where necessary. For each of these criteria, one of the following assessments will be given:

- Low risk of bias: plausible bias unlikely to alter results.
- Unclear risk of bias: plausible bias that raises some doubt about the results.
- High risk of bias: plausible bias that seriously weakens confidence in results.

In order to ensure a standardized assessment of risk of bias, we will conduct an in-person calibration process, in which we will assess and discuss multiple studies.

### *Dealing with missing data*

We will contact the authors of included studies for which data related to study methods, participants, outcome data, or statistics are unclear or missing. If estimates for the full population are not provided (e.g. estimates for men and women reported separately), we will calculate them using the data available, request additional data from authors where calculation is not possible, or simply report what is reported in the study, without any imputation and subsequent calculation. We will take note of all missing outcome data in the data extraction form and consider these during the risk of bias assessment.

### *Measurement of treatment effect*

To facilitate comparability across the observed effects of included studies, we will aim to report all effects using common metrics: risk ratios (RR) for dichotomous outcomes and mean differences (MDs) for continuous outcomes. We will also report 95% confidence intervals where possible. For studies that report alternative measures and/or do not report 95% confidence intervals, we will attempt to calculate RRs or MDs with 95% confidence intervals, where possible.

### *Evidence synthesis*

For each primary outcome and each intervention category (road environment, road user behavior, legislation and enforcement and vehicle design and condition), where two or more studies of similar design assess populations, settings, interventions and outcomes that are homogenous enough to enable meaningful comparison, we will conduct random-effects meta-analysis. However, based on the expected heterogeneity of the identified participants, interventions, comparisons, outcomes, contexts, and the methodologies used to evaluate these interventions, meta-analysis may not be appropriate. Therefore, where meta-analysis is not conducted, we will present findings both in summary tables and graphically through harvest plots. The harvest plot has been shown to be an effective, clear and transparent way to portray evidence from a heterogeneous evidence base, especially where primary studies are not well-suited to statistical pooling.^17-19^ It is a matrix-based, flexible graphical approach, allowing the systematic reviewer to decide which characteristics will define the X- and Y-axes. A separate harvest plot will be created for each intervention category. We will arrange each study, represented by a bar, in rows according to the assessed outcome; and in columns according to the direction of effect - favoring control, no difference, and favoring intervention. Additionally, the height of the bar represents the study design, with RCTs being the highest bar, followed by nonrandomized controlled trials.

### *Subgroup analyses*

Where sufficient data are present, we will also use either meta-analysis or harvest plots to assess whether certain factors may have influenced intervention effectiveness. Factors of interest include:

- Age
  - Children
  - Adults
- Setting
  - Urban
  - Rural
- Injury severity
- Global Burden of Disease (GBD) super region.^20^
  - Central Europe, Eastern Europe and Central Asia,
  - Latin America and Caribbean
  - North Africa and Middle East
  - Southeast Asia, East Asia and Oceania
  - South Asia
  - Sub-Saharan Africa
  - High income

### *Assessment of certainty of evidence*

In order to assess the overall certainty of the evidence of assessed comparisons, we will grade the certainty of evidence using the Grading of Recommendations Assessment, Development and Evaluation (GRADE) system. We will prepare a ‘Summary of findings’ tables for all primary outcomes for the relevant comparisons under each intervention category.

For GRADE, two reviewers will assess the overall certainty of the evidence by considering five factors for downgrading the certainty (risk of bias, inconsistency, indirectness, imprecision, publication bias), and three factors for upgrading the certainty (large effect size, all plausible confounding would reduce the demonstrated effect, dose response gradient). For each GRADE factor, we will provide a judgment with rationale included as a footnote in the Summary of Findings table. The outcomes presented in the ‘Summary of findings’ will be:

- Pedestrian crashes
- Pedestrian injuries
- Pedestrian hospitalizations
- Pedestrian deaths

### *Sensitivity analysis*

In order to assess whether decisions regarding the review methodology influenced the results of the review, we will conduct a series of sensitivity analyses. We will stratify all analyses based on study design, separating study designs based on the inherent internal validity associated with the design, thus randomized studies, followed by c-ITS studies, and finally CBA and ITS studies. We will additionally assess the robustness of the review findings to risk of bias by conducting the evidence synthesis without any studies judged to have a serious risk of bias.

# Acknowledgements

This work is funded through a grant (# VN 81204847) by the German Federal Ministry of Education and Research (BMBF).

We thank the following persons who have participated in various aspects of the protocol development, including piloting the study search strategy: Stella Namatovu, Albert Ningwa, and Frederick Oporia. We thank Lisa Pfadenhauer for her important input during early discussions of the review scope.

# Declarations of interest: No competing interests

# References

1. Lozano R, Naghavi M, Foreman K, et al. Global and regional mortality from 235 causes of death for 20 age groups in 1990 and 2010: a systematic analysis for the Global Burden of Disease Study 2010. *The lancet.* 2013;380(9859):2095-2128.

2. World Health Organization. *The global burden of disease: 2004 update.* Geneva: World Health Organization;2008.

3. World Health Organization. *Status Report on Road Safety 2013: Supporting a Decade of Action. .* Geneva: World Health Organization;2013.

4. M G, GR M, G D, H T. Regulatory and road engineering interventions for preventing road traffic injuries and fatalities among vulnerable (non-motorised and motorised two-wheel) road users in low- and middle-income countries | Cochrane. *Cochrane.* 2015.

5. Haddon W, Jr. Energy damage and the 10 countermeasure strategies. 1973. *Inj Prev.* 1995;1(1):40-44.

6. Haddon W, Jr. On the escape of tigers: an ecologic note. *Am J Public Health Nations Health.* 1970;60(12):2229-2234.

7. Runyan CW. Using the Haddon matrix: introducing the third dimension. *Inj Prev.* 1998;4(4):302-307.

8. Hazen A, Ehiri JE. Road traffic injuries: hidden epidemic in less developed countries. *Journal of the National Medical Association.* 2006;98(1):73.

9. Higgins J, Green S. *Cochrane Handbook for Systematic Reviews of Interventions*  Version 5.1.0 [updated March 2011]. The Cochrane Collaboration. In:2011.

10. Staton C, Vissoci J, Gong E, et al. Road Traffic Injury Prevention Initiatives: A Systematic Review and Metasummary of Effectiveness in Low and Middle Income Countries. *PLoS One.* 2016;11(1):e0144971.

11. Bunn F, Collier T, Frost C, Ker K, Roberts I, Wentz R. Area-wide traffic calming for preventing traffic related injuries. *Cochrane Database Syst Rev.* 2003(1):Cd003110.

12. Wazana A, Krueger P, Raina P, Chambers L. A review of risk factors for child pedestrian injuries: are they modifiable? *Inj Prev.* 1997;3(4):295-304.

13. Cochrane. EPOC resources for review authors. 2013; <http://epoc.cochrane.org/resources/epoc-resources-review-authors>. Accessed 6/25, 2018.

14. Sterne JA, Hernan MA, Reeves BC, et al. ROBINS-I: a tool for assessing risk of bias in non-randomised studies of interventions. *Bmj.* 2016;355:i4919.

15. WHO. *Pedestrian safety: a road safety manual for decision-makers and practitioners.* World Health Organization.

16. Tugwell P, Petticrew M, Kristjansson E, et al. Assessing equity in systematic reviews: realising the recommendations of the Commission on Social Determinants of Health. *Bmj.* 2010;341:c4739.

17. Ogilvie D, Fayter D, Petticrew M, et al. The harvest plot: a method for synthesising evidence about the differential effects of interventions. *BMC Med Res Methodol.* 2008;8:8.

18. Turley R, Saith R, Bhan N, Doyle J, Jones K, Waters E. Slum upgrading review: methodological challenges that arise in systematic reviews of complex interventions. *J Public Health (Oxf).* 2013;35(1):171-175.

19. Nehring I, Kostka T, von Kries R, Rehfuess EA. Impacts of in utero and early infant taste experiences on later taste acceptance: a systematic review. *J Nutr.* 2015;145(6):1271-1279.

20. Gakidou E, Afshin, A., Abajobir, A. A. Global, regional, and national comparative risk assessment of 84 behavioural, environmental and occupational, and metabolic risks or clusters of risks, 1990-2016: a systematic analysis for the Global Burden of Disease Study 2016. *Lancet.* 2017;390(10100):1345-1422.

# Appendix 1 – MEDLINE search strategy

| Search  component | | Searches | | Results |
| --- | --- | --- | --- | --- |
| Population | 1 | | (pedestrian* or pietons* or vulnerable road user* or VRU*non-motori* or non motorist or non motorists or safety).mp | 960358 |
| Intervention | 2 | | (road* or street* or traffic or vehicle*).ti,ab. | 253914 |
| Outcome | 3 | | (accident* or crash* or collision* or injur* or wound* or mortality or death* or fatal* or hospitalization* or hospitalisation* or hospital admission or hospital admissions).ti,ab | 3006616 |
| PIO combined | 4 | | 1 and 2 and 3 | 11529 |
| Study design | 5 | | randomized controlled trial/ or randomized/ or controlled study/ or comparative study/ or clinical study/ or quasi experimental study/ or experimental study/ or control group/ or follow up/ or prospective study/ or retrospective study/ | 8331430 |
| … | 6 | | (randomized or placebo or (random* and trial* and group*)).mp. | 1191798 |
| … | 7 | | (control group* or follow-up stud* or follow-up assessment or prospectiv* or non-random*or nonrandom*).mp. | 1576998 |
| … | 8 | | (before after stud* or "before and after" or time series or time-series or retrospective* or longitud* or (controlled and cohort* and stud*)).mp. | 1971894 |
| … | 9 | | (controlled before or pre test or pretest or posttest or post test or pre intervention or post intervention).mp. | 54228 |
| … | 10 | | or/5-9 | 9724847 |
| PIOS combined | 11 | | 4 and 10 | 4146 |

Table 2: Ovid search strategy developed for use in MEDLINE.

# Appendix 2 – Title/abstract screening form

Guidance for title/abstract screening for systematic review:

**Interventions to prevent road traffic injuries and deaths among pedestrians: a protocol for a global systematic review**

Review objective: To assess the effectiveness of interventions to reduce pedestrian injuries and deaths.

Guidance: This document provides guidance on the title/abstract screening stage of the review. As outlined in the protocol, all titles and abstracts will be screened independently and in duplicate (i.e. by two reviewers) against our inclusion criteria to determine eligibility for inclusion. In order to be eligible, a study must match criteria related to the population, intervention, comparison and outcomes (PICO), as well as the study design. **It is important at this stage, to be inclusive rather than exclusive**, i.e. for any potentially eligible or unclear cases, their full texts will be retrieved and then assessed for eligibility in duplicate.

The PICO(S) eligibility criteria, described in detail in the protocol, are outlined in Table 3 below. You may use this form to help guide screening titles and abstracts but you **do not need to complete** the form for any study screened at this stage.

We will use EndNote software to manage retrieved studies and to remove duplicate reports of the same study and the online software Rayyan to screen titles and abstracts: <https://rayyan.qcri.org/welcome>. You will need to create an account; access is free.

In Rayyan:

- As you come across a title/abstract to screen you will have to select whether to **include**, **exclude**, or whether it is **undecided** (if it is unclear whether it meets all the eligibility criteria, i.e. if there is not enough information).
- Some records have been marked as duplicates by Rayyan – please leave these to one of the senior authors to resolve and screen those records that are not marked as duplicates.

**Table 3.** Review eligibility criteria

| Population & setting | We will include studies assessing the effect of interventions directly or indirectly impacting pedestrians of any age and setting (HICs, MICs, or LICs). We will define pedestrians as per the World Health Organization guidelines. |
| --- | --- |
|  | **Exclusion criteria:**   - Modeling studies, simulation and qualitative studies will be excluded. |
| Intervention  We will **include** four categories of **road safety** interventions that directly or indirectly reduce pedestrian injuries and deaths | **(1) Road environment** (e.g. traffic calming features (e.g. speed humps and rumble strips), separation of vulnerable road users from fast motorized traffic, and street lighting and visibility enhancement) |
|  | **(2) Road user behavior** (pedestrian education and behavioral change interventions) |
|  | **(3) Legislation and enforcement** (e.g. laws and regulations on alcohol and mobile phone use while driving, licensing laws, and vehicle inspection laws). |
|  | **(4) Vehicle design and road worthiness status** (e.g. frontal features protective of pedestrians). |
|  | **Exclusion criteria:**   - Interventions solely targeting other road users and no indirect effect on pedestrian safety (e.g. helmet laws) - Post-crash interventions (e.g. pre-hospital emergency and rehabilitative services) |
| Outcome  Studies assessing any of the listed primary outcomes will be included.  **Note**: **do NOT exclude** studies based on whether they report these outcomes at this title/abstract screening stage | **Primary outcomes**   1. Pedestrian crashes 2. Pedestrian injuries 3. Pedestrian hospitalizations 4. Pedestrian deaths |
|  | **Secondary outcomes**   1. Costs of pedestrian crashes and injuries 2. Number or proportion of pedestrian disabilities 3. Number of arrests for drunk driving 4. Number of speeding tickets 5. Change in pedestrian knowledge (for educational interventions) |
|  | **Adverse outcomes**   1. Any reports of adverse effects (e.g. displacement of accidents and/or traffic volumes to other parts/surrounding road networks, slow moving traffic, and traffic congestion at intersections). |
| Study design  We will include the listed randomized and selected non-randomized study designs  **Note**: **do NOT exclude** studies based on study design at this stage – determining study design from the abstract is often not possible | - **Randomized controlled trials (RCTs):** Experimental studies in which people are randomly allocated to intervention or control group; - **Cluster randomized controlled trials (c-RCTs):** Experimental studies in which clusters (e.g. study groups or study sites) are allocated to intervention or control using methods of randomization; - **Controlled before-after (CBA) studies**: Studies in which observations are made before and after the implementation of an intervention, both in a group that receives the intervention and in a control group that does not. CBA studies must have a minimum of one intervention and one control site; - **Interrupted time-series (ITS) studies**: Studies that use observations at multiple time points before and after an intervention. ITSs can be either uncontrolled (u-ITS) or controlled (c-ITS); they must have at least three data points before and after a clearly defined intervention (in terms of content and timing). |
| Language & publication status | We will include studies in any language and regardless of their publication status and year of publication. |

# Appendix 3 – Full text screening form

| **STUDY ELIGIBILITY SCREENING STRATEGY** | | | |
| --- | --- | --- | --- |
|  | | | |
| Study ID: | | | |
| Review title: | | | |
| Review objective: To assess the effectiveness of interventions to reduce pedestrian injuries and deaths. | | | |
|  | | | |
| **Duplicate** | | | |
| Is this the same title/abstract as one that you screened before? | Yes ↓ | | No ↓ |
|  | **Tag as "Duplicate" and then stop here** | | Go to next question |
| **Title/Abstract Language** | | | |
| Is the title/abstract written in English | Yes ↓ | | No ↓ |
|  | Go to next question | | **Tag as "No English TIAB" and go to next question** |
| Is the title/abstract written in a language that you can read and understand?  Or is the title/abstract written in any of the following languages: Spanish, Italian, French or German. | Yes ↓ | | No ↓ |
|  | Go to next question | | **Tag as “Cannot read” and then stop here** |
| **Type of study** | | | |
| Is the study an RCT, cluster RCT, CBA, or ITS study?  • *Randomized controlled trials (RCTs)*: Experimental studies in which people are randomly allocated to intervention or control group;  • *Cluster randomized controlled trials (c-RCTs):* Experimental studies in which clusters (e.g. study groups or study sites) are allocated to intervention or control using methods of randomization;  • *Controlled before-after (CBA) studies*: Studies in which observations are made before and after the implementation of an intervention, both in a group that receives the intervention and in a control group that does not – **studies must assess** **at least one intervention and one control site**;  • *Interrupted time-series (ITS) studies*: Studies that use observations at multiple time points before and after an intervention in one population. ITS studies may also include a control group (cITS study) – **studies must have at least three data points before and after a clearly defined intervention (in terms of content and timing).** | Yes ↓ | Unclear ↓ | No ↓ |
|  | Go to next question | | **EXCLUDE: * provide reason as =ineligible study design** |
| **Type of population** | | | |
| Included:   - Pedestrians - Other road users targeted with interventions that indirectly impact pedestrians - Mixed population study (**if mixed population, include only if outcomes specifically for pedestrians are reported**)   Excluded:   - Other road users targeted with interventions that do not indirectly impact pedestrians | Yes ↓ | Unclear ↓ | No ↓ |
|  | Go to next question | | **EXCLUDE: * provide reason as =ineligible participants** |
| **Setting** | | | |
| Is the study conducted in a LIC? | Yes ↓ | Unclear ↓ | No ↓ |
|  | Tag as **LIC** and Go to next question | Tag as **Unclear setting** and go to next question | Tag as **MIC** or **HIC** and go to next question |
| Is the study conducted in a MIC? | Yes ↓ | Unclear ↓ | No ↓ |
|  | Tag as **MIC** and Go to next question | Tag as **Unclear setting** and go to next question | Tag as **HIC** and go to next question |
| **Type of intervention** | | | |
| Is the study reporting on:  (1) Road environment interventions? e.g.   - Traffic calming interventions (e.g. traffic lights and speed humps) - Separation of vulnerable road users from fast motorized traffic - Street lighting and visibility enhancement, etc.   (2) Legislation and enforcement interventions? e.g.   - Alcohol use laws and regulations - Mobile phone use laws and regulations - Licensing laws - Vehicle inspection laws, etc.   (3) Road user behavior change interventions? e.g.   - Education and behavioral change campaigns - Pedestrian visibility enhancement (e.g. bright clothes), etc.   or  (4) Vehicle interventions? e.g.   - Frontal features protective of pedestrians, etc. | Yes ↓ | Unclear ↓ | No ↓ |
|  | Go to next question | | **EXCLUDE: * provide reason as =ineligible intervention** |
| **Outcomes** | | | |
| Which of the following outcomes did they measure?  (Highlight the relevant outcomes)  Primary   - Pedestrian crashes - Pedestrian injuries - Pedestrian hospitalizations - Pedestrian deaths   Secondary   - Costs of pedestrian crashes and injuries - Number or proportion of pedestrian disabilities - Number of arrests for drunk driving - Number of speeding tickets - Change in pedestrian knowledge (for educational interventions) |  |  |  |
| **Proceed to final decision on inclusion or exclude, and give reason as other, with details** | **Include** | **Exclude**  Reason for Exclusion:  ________________________  ____________________________ | |
| Any other comments?  ______________________________________________________________________________________________________  ________________________________________________________________________________________________________  _______________________________________________________________________________________________________ | | | |

*** providing a reason is only valid for full-text screening**
